# Natural variation in a single amino acid substitution underlies physiological responses to topoisomerase II poisons

**DOI:** 10.1371/journal.pgen.1006891

**Published:** 2017-07-12

**Authors:** Stefan Zdraljevic, Christine Strand, Hannah S. Seidel, Daniel E. Cook, John G. Doench, Erik C. Andersen

**Affiliations:** 1 Interdisciplinary Biological Sciences Program, Northwestern University, Evanston, Illinois, United States of America; 2 Department of Molecular Biosciences, Northwestern University, Evanston, Illinois, United States of America; 3 Broad Institute of MIT and Harvard, Cambridge, Massachusetts, United States of America; 4 Biology Department, Eastern Michigan University, Ypsilanti, Michigan, United States of America; 5 Robert H. Lurie Comprehensive Cancer Center of Northwestern University, Chicago, Illinois, United States of America; University of Kansas, UNITED STATES

## Abstract

Many chemotherapeutic drugs are differentially effective from one patient to the next. Understanding the causes of this variability is a critical step towards the development of personalized treatments and improvements to existing medications. Here, we investigate sensitivity to a group of anti-neoplastic drugs that target topoisomerase II using the model organism *Caenorhabditis elegans*. We show that wild strains of *C*. *elegans* vary in their sensitivity to these drugs, and we use an unbiased genetic approach to demonstrate that this natural variation is explained by a methionine-to-glutamine substitution in topoisomerase II (TOP-2). The presence of a non-polar methionine at this residue increases hydrophobic interactions between TOP-2 and its poison etoposide, as compared to a polar glutamine. We hypothesize that this stabilizing interaction results in increased genomic instability in strains that contain a methionine residue. The residue affected by this substitution is conserved from yeast to humans and is one of the few differences between the two human topoisomerase II isoforms (methionine in hTOPIIα and glutamine in hTOPIIβ). We go on to show that this amino acid difference between the two human topoisomerase isoforms influences cytotoxicity of topoisomerase II poisons in human cell lines. These results explain why hTOPIIα and hTOPIIβ are differentially affected by various poisons and demonstrate the utility of *C*. *elegans* in understanding the genetics of drug responses.

## Author summary

The severe cytotoxic effects associated with anti-neoplastic treatment regimens make it difficult to assess the contributions of genetic variation on treatment responses in clinical settings. Therefore, we leveraged genetic diversity present in the metazoan model nematode *Caenorhabditis elegans* to identify genetic variants that contribute to differential susceptibility to a broadly administered class of anti-neoplastic compounds that poison the activity of topoisomerase II enzymes. We show that wild *C*. *elegans* isolates contain either glutamine or methionine at a highly conserved residue of the topoisomerase II (TOP-2) protein and that this substitution is predictive of animal responses to the topoisomerase II poisons etoposide, teniposide, dactinomycin, and XK469. Interestingly, the two human versions of this protein, hTOPIIα and hTOPIIβ, contain a methionine or glutamine at the corresponding residue, respectively. We show that this difference between the two human topoisomerase II isoforms contributes to the differential cytotoxicity induced by these drugs. Taken together, our results highlight the power of studying the effects of natural genetic variation on drug responses in a model organism and propose methods to develop new drugs that have increased affinity for the desired hTOPIIα isoform expressed in tumor cells.

## Introduction

Antineoplastic regimens used to treat cancer are often associated with poor prognoses and severe side effects. Ideally, antineoplastic regimens can be tailored to an individual patient based on various genetic markers known to be associated with drug response to maximize therapeutic effectiveness and minimize unwanted side effects. Advances in sequencing technologies over the course of the past decade promised the discovery of many genetic variants that contribute to human health. Though large-scale sequencing projects have led to the identification of many genetic variants associated with disease risk [1], relatively few variants have been identified that contribute to clinically relevant traits such as response to antineoplastic compounds. In fact, only 71 of over 500 FDA-approved antineoplastic compounds use genetic information to affect treatment efficacy (www.fda.gov). Unfortunately, the predictive power of these identified genetic variants can be inconsistent due to biases in the sampled population [2] and other key limitations of clinical genome-wide association (GWA) studies that attempt to link genetic variants with treatment outcomes. The major factor limiting the efficacy of these studies is sample size because it is difficult to identify large numbers of individuals exposed to the same antineoplastic regimens. This limitation is compounded when considering environmental [3,4] and tumor heterogeneity [5]. As a result, most variants discovered to be associated with outcomes in clinical GWA studies offer low predictive power for patient responses to treatment [6]. These limitations and others emphasize the need for novel approaches to identify variants that predict patient outcomes to antineoplastic compounds.

Studies of model organisms have greatly facilitated our understanding of basic cellular processes. In recent years, *Saccharomyces cerevisiae* and *Drosophila melanogaster* have been used to understand the physiological effects of small molecules and repurposed as screening platforms to identify new antineoplastic compounds [7–9]. The ability to generate extremely large numbers of recombinant yeast facilitates the identification of genomic regions that are predictive of drug response [10,11]. Furthermore, the specific genes and variants within regions can be identified and functionally validated in yeast [12–14]. By contrast, *D*. *melanogaster* studies offer the ability to study the physiological responses to drugs in the context of multiple tissue types, but functional validation of specific genes and variants associated with drug responses has been more limited [9]. The roundworm *Caenorhabditis elegans* has the advantages of both *S*. *cerevisiae* and *D*. *melanogaster* because large cross populations can be generated to study the physiological responses to drugs in a metazoan. These attributes have made *C*. *elegans* an important model for connecting differential drug responses with genetic variants present in the species [15,16].

Here, we take advantage of natural genetic variation present in *C*. *elegans* to identify the genetic basis underlying susceptibility to a panel of clinically relevant antineoplastic compounds that poison the activity of topoisomerase II enzymes. The inhibition of these enzymes by topoisomerase II poisons results in the accumulation of double-stranded breaks and genome instability [17–19]. Topoisomerase II enzymes are targeted by antineoplastic regimens because proliferative cell populations require their enzymatic activity to relieve topological stress ahead of the replication fork [20]. Using two unbiased genetic mapping approaches, we show that divergent physiological responses to the topoisomerase II poison etoposide are determined by natural genetic variation in a *C*. *elegans* topoisomerase II enzyme. Furthermore, we show using CRISPR/Cas9-mediated genome editing that variation in a specific amino acid (Q797M) underlies the observed phenotypic variation in response to etoposide. This residue is conserved in humans and is one of the few differences between the putative drug-binding pockets of the two topoisomerase II isoforms (M762 in hTOPIIα and Q778 in hTOPIIβ). Previous structural studies on hTOPIIβ implicated this glutamine residue in etoposide binding because of its proximity to the drug-binding pocket [21,22]. However, a study on hTOPIIα suggested that the corresponding methionine residue has no functional role in drug binding [23]. We present a mechanistic model to explain how variation at this residue underlies differential responses to etoposide and other topoisomerase II poisons. Finally, we use genome-edited human cell lines to show that this residue in hTOPIIα contributes to differential toxicity of various topoisomerase II poisons. These results demonstrate the power of using *C*. *elegans* natural genetic variation to identify mechanisms of drug susceptibility in human cells that could inform human health decisions based on genetic information.

## Results

### A single major-effect locus explains variation in response to etoposide

We investigated etoposide sensitivity in *C*. *elegans* using a high-throughput fitness assay. In brief, animals were grown in liquid culture in presence of etoposide, and body lengths of progeny and offspring production were measured using a COPAS BIOSORT (S1 Fig). In this assay, shorter body lengths are indicative of developmental delay. To identify an appropriate dose of etoposide for this assay, we performed dose-response experiments on four genetically diverged isolates of *C*. *elegans*: N2 (Bristol), CB4856 (Hawaii), JU258, and DL238. We chose 250 μM etoposide for further experiments because it was the lowest concentration at which we observed an etoposide-specific effect in all four strains tested, trait differences between the laboratory Bristol strain (N2) and a wild strain from Hawaii (CB4856) strains were maximized, and the median animal length was highly heritable (S2 Fig).

**Fig 1:**
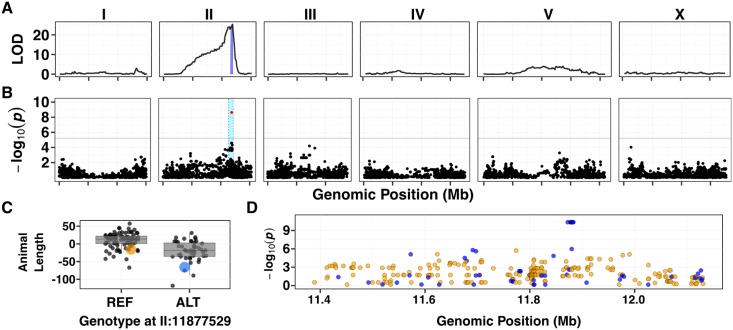
A major-effect locus controls differences in etoposide response. (A) Linkage mapping plot for regressed median animal length in the presence of etoposide is shown. The significance values (logarithm of odds, LOD, ratio) for 1454 markers between the Bristol and Hawaiian strain are on the y-axis, and the genomic position (Mb) separated by chromosome is plotted on the x-axis. Each tick on the x-axis corresponds to 5 Mb. The associated 1.5 LOD-drop confidence intervals are represented by blue bars. (B) A manhattan plot for regressed median animal size in the presence of etoposide is shown. Each dot represents an SNV that is present in at least 5% of the phenotyped population. The –*log*_10_(*p*) for each SNV is plotted on the y-axis, and the genomic position (Mb) is plotted on the x-axis. Each tick on the x-axis corresponds to 5 Mb. SNVs are colored red if they pass the genome-wide Bonferroni-corrected significance threshold, which is denoted by the gray horizontal line. The genomic region of interest surrounding the QTL is represented by a cyan rectangle. (C) Tukey box plots of phenotypes used for association mapping in (B) are shown. Each dot corresponds to the phenotype of an individual strain, which is plotted on the y-axis. Strains are grouped by their genotype at the peak QTL position (red SNV from panel B, chrII:11877529), where REF corresponds to the allele from the reference Bristol strain. The Bristol and Hawaii strains are colored orange and blue, respectively. (D) Fine mapping of the chromosome II region of interest, showing the calculated *p*-value associated with the Spearman's *rho* statistic for every moderate to severe effect variant, as predicted by snpeff, present in the QTL confidence interval from (B), is shown. The genomic position of each SNV is on the x-axis in Mb and the *p*-value associated with the Spearman’s *rho* statistic is on the y-axis. Blue dots correspond to variants that are present in the Hawaiian strain, and orange dots correspond to variants that are present in the wild population.

When grown in etoposide, the progeny of both the Bristol and Hawaii strains are likely developmentally delayed and therefore shorter in animal length. However, the median of the Hawaii strain is on average 60 μm (or 31%) shorter than progeny of the Bristol strain because they are more severely affected by etoposide. To map the genetic variants underlying this difference, we performed our high-throughput fitness assay on a panel of 265 recombinant inbred advanced intercross lines (RIAILs), generated between a Bristol derivative (QX1430) and Hawaii [16]. We measured median animal length for each RIAIL strain grown in etoposide, and we corrected for assay-to-assay variability and effects of the drug carrier (DMSO) using a linear model. We used the resulting regressed median animal length trait (referred to as animal length) for quantitative trait locus (QTL) mapping. This mapping identified a major-effect QTL for etoposide resistance on chromosome II at 11.83 Mb (Fig 1A). This QTL explained 27% of the phenotypic variance among the recombinant lines. The QTL confidence interval spans from 11.67 to 11.91 Mb on chromosome II and contains 90 genes, 68 of which contain variation between the parental strains.

We next sought to validate this QTL using homozygous reciprocal near-isogenic lines (NILs), which contain either the QTL confidence interval from the Bristol strain introgressed into the Hawaii strain or the interval from the Hawaii strain introgressed into the Bristol strain. NILs with the genomic interval derived from the Bristol strain have increased resistance to etoposide compared to the Hawaii strain (S3 Fig). Similarly, NILs with the genomic interval derived from the Hawaii strain exhibited decreased resistance to etoposide. These results confirmed that genetic variation located on the right arm of chromosome II contributes to differential etoposide susceptibility.

### The same locus on chromosome II explains variation in response to etoposide in a panel of wild *C*. *elegans* isolates

In the initial dose response experiments, we found that JU258 and DL238 had different responses to etoposide than the Bristol and Hawaii strains, suggesting that additional genetic variation present in the wild *C*. *elegans* population could also contribute to etoposide response. To identify this additional variation, we performed a genome-wide association (GWA) mapping of etoposide resistance in 138 wild *C*. *elegans* isolates. This analysis led to the identification of a QTL on the right arm of chromosome II with a peak position at 11.88 Mb (Fig 1B). This QTL has a genomic region of interest that spans from 11.70 to 12.15 Mb for which we found no evidence of selection (S4 Fig) or geographic clustering of the peak QTL allele (S5 Fig). In addition, this QTL overlaps with the QTL identified through linkage mapping described above. Of the 138 wild isolates assayed, including the Hawaiian strain, 46 have the alternate (non-Bristol) genotype at the peak position on chromosome II (Fig 1C). Similar to our observations using the recombinant lines, the 46 strains that contain the alternate genotype are more sensitive to etoposide than strains containing the Bristol genotype at the QTL peak marker. We hypothesized that variation shared between the Hawaiian strain and the other 45 alternate-genotype strains contributes to etoposide sensitivity because we detected overlapping QTL, with the same direction of effect, between GWA and linkage mapping experiments. This hypothesis suggested that we could condition a fine-mapping approach on variants found in the Hawaiian strain and shared across these 45 strains.

To fine-map the QTL, we focused on variants shared among wild isolates. Using data from the *C*. *elegans* whole-genome variation dataset [24] we calculated the *p*-value of Spearman's *rho* correlation coefficients between animal length and each single-nucleotide variant (SNV) in the QTL confidence interval (Fig 1D). SNVs in only three genes, *npp-3*, *top-2*, and *ZK930*.*5*, were highly correlated with the etoposide response (-log10(*p*) = 10). Of these genes, the *top-2* gene encodes a topoisomerase II enzyme that is homologous to the two human isoforms of topoisomerase II. We prioritized *top-2* because topoisomerase II enzymes are the cellular targets for etoposide [17].

### Genetic variation in *top-2* contributes to differential etoposide sensitivity

**Fig 2:**
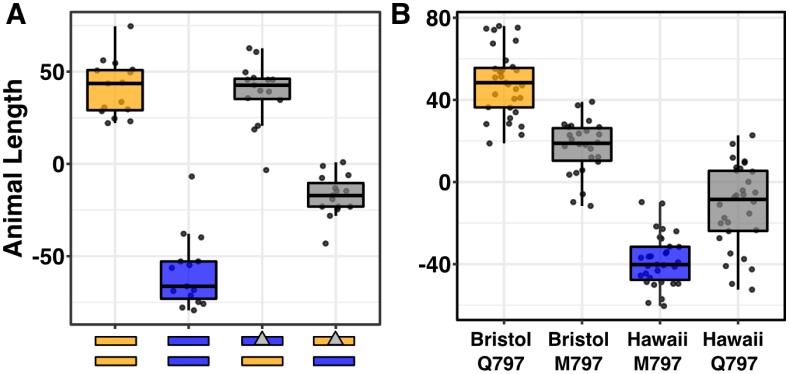
Q797M variant in TOP-2 underlies etoposide sensitivity in *C*. *elegans*. (A) Tukey box plots of the residual median animal length distribution of Bristol (orange) and Hawaii (blue) compared to two heterozygous *top-2* deletion strains (gray) in response to etoposide are shown. Orange (Bristol) and blue (Hawaii) rectangles below the plot correspond to the two chromosome II homolog genotypes. Gray triangles denote chromosomes with the *top-2* deletion allele. The Bristol strain and the Bristol/Hawaii(Δ*top-2*) heterozygous strain are not significantly different from each other (Tukey’s HSD, *p*-value 0.9990812), but all other comparisons are significant (Tukey’s HSD *p*-value < 0.0001). (B) Tukey box plots of residual median animal length after etoposide exposure are shown (Bristol, orange; Hawaii, blue; allele replacement strains, gray). Labels correspond to the genetic background and the corresponding residue at position 797 of TOP-2 (Q for glutamine, M for methionine). Every pair-wise strain comparison is significant (Tukey’s HSD, Bristol—Hawaii, Bristol—Hawaii swap, Hawaii—Bristol swap *p*-value < 2.0E-14; Bristol—Bristol swap *p*-value = 1.2E-10; Bristol swap—Hawaii swap *p*-value = 3.4E-9; Hawaii—Hawaii swap *p*-value = 1.3E-8).

To determine if genetic variation present in the *top-2* gene contributes to differential etoposide sensitivity, we performed a reciprocal hemizygosity test [14]. Prior to this test, we determined that resistance to etoposide is dominant by measuring the lengths of F1 heterozygotes from a cross between the Bristol and Hawaii strains in the presence of etoposide (S6 Fig). Additionally, we tested *npp-3* and *top-2* deletion alleles from the Bristol genetic background and found that only loss of *top-2* contributes to etoposide sensitivity (S7 Fig). To more definitively show a causal connection of *top-2* variation to etoposide sensitivity, we used a reciprocal hemizygosity test. First, we introgressed the *top-2(ok1930)* deletion allele into the Hawaiian genetic background. The Bristol/Hawaii(Δ*top-2*) heterozygote that contains the Bristol *top-2* allele is more resistant to etoposide treatment than the Hawaii/Bristol(Δ*top-2*) heterozygote, which suggests that the Bristol *top-2* allele underlies etoposide resistance (Fig 2A, S8 Fig). The observed differences between the Hawaii/Bristol(Δ*top-2*) and Bristol/Hawaii(Δ*top-2*) heterozygotes confirmed that *top-2* variation underlies differential susceptibility to etoposide.

### A glutamine-to-methionine variant in TOP-2 contributes to etoposide response

To identify genetic variants in *top-2* that contribute to etoposide resistance in the Bristol strain, we focused on genomic differences between the Bristol and Hawaii strains. Based on gene expression data between the Bristol and Hawaii strains [25], *top-2* is expressed at similar levels. Therefore, we concluded that etoposide resistance in the Bristol strain is likely caused by coding variation. The *C*. *elegans top-2* gene contains 31 SNVs across the population-wide sample of 138 wild isolates. We narrowed our search to 16 variants present in the Hawaiian strain. Two of these variants are in the 3' UTR, three are in introns, and six are synonymous variants that likely do not contribute to etoposide resistance. The remaining five variants encode for amino acid changes in the TOP-2 enzyme. Of these five variants, four were highly correlated with etoposide sensitivity in the wild isolate panel: Q797M, I1206L, Q1217A, and D1387N. Multiple-sequence alignment of TOP-2 peptides across yeast, *D*. *melanogaster*, mice, and humans revealed that I1206L, Q1217A, and D1387N are in the variable C-terminal domain (S1 File). By contrast, the Q797M variant is located in the conserved DNA binding and cleavage domain [26]. Structural data from human orthologs suggest that the TOP-2 Q797 residue lies within the putative etoposide-binding pocket [21], and the corresponding residue is a methionine (M762) in the hTOPOIIα and a glutamine (Q778) in hTOPOIIβ [23]. Additionally, the two human isoforms differ in one other residue within the putative etoposide-binding pocket (S800(α),A816(β)). Therefore, the *C*. *elegans* glutamine-to-methionine TOP-2 variant mirrors one of two differences within the etoposide-binding pocket of the two human topoisomerase II enzyme isoforms. Crucially, hTOPOIIα forms a more stable DNA-TOPOII cleavage complex with etoposide than hTOPOIIβ [27]. We hypothesized that etoposide sensitivity in both *C*. *elegans* and the human isoforms is affected by this residue.

To test the effects of the Q797M variant on *C*. *elegans* response to etoposide, we used CRISPR/Cas9-mediated genome editing to change this residue. We replaced the glutamine residue in the Bristol strain with a methionine and the methionine residue in the Hawaii strain with a glutamine. We exposed the allele-replacement strains to etoposide and found that the methionine-containing Bristol animals were more sensitive than glutamine-containing Bristol animals (Fig 2B). Conversely, the glutamine-containing Hawaii animals were more resistant to etoposide than the methionine-containing Hawaii animals (Fig 2B). These results are consistent with the linkage mapping experiments because the allele-replacement strains account for approximately 25% of the phenotypic variation between the Bristol and Hawaiian strains and other loci could contribute to etoposide response.

### Methionine mediates stronger hydrophobic interactions with etoposide than glutamine

**Fig 3:**
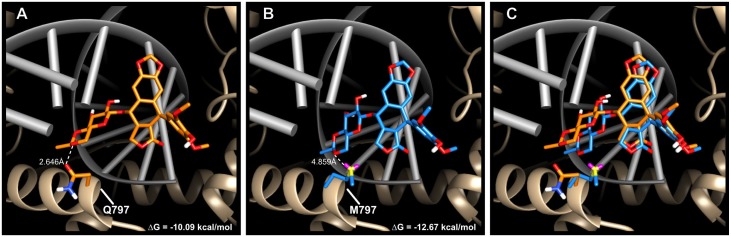
Etoposide docked into the *C*. *elegans* TOP-2 homology model. Etoposide docked into the (A) glutamine-containing Bristol TOP-2 enzyme (Δ*G* = -10.09 kcal/mol), (B) methionine-containing Hawaii TOP-2 enzyme (Δ*G* = -12.67 kcal/mol), and (C) the overlay of both structures. Glutamine is colored in orange, and methionine is colored in blue. Etoposide docked into the glutamine-containing TOP-2 enzyme is orange, and etoposide docked into the methionine-containing TOP-2 enzyme is blue. DNA is colored in gray, and the ribbon representation of the TOP-2 protein is shown in tan.

We hypothesized that the non-polar functional group attached to the glycosidic bond of etoposide contributes to increased stability of the drug-enzyme complex by forming a more stable interaction with the methionine residue than with the glutamine residue. To test this hypothesis, we simulated etoposide docking into the putative drug-binding pocket of the TOP-2 homology model generated by threading the *C*. *elegans* peptide sequence into the hTOPOIIβ structure (RMSD = 1.564Å, PDB:3QX3; [21]). Upon etoposide binding, the free energy (Δ*G*) of the drug-binding pocket was -10.09 kcal/mol for TOP-2 Q797 (Fig 3A) and -12.67 kcal/mol for TOP-2 M797 (Fig 3B). This result suggests that etoposide interacts more favorably with TOP-2 M797 than with TOP-2 Q797, consistent with our results in live animals. A more favorable drug-enzyme interaction, as indicated by a more negative Δ*G*, likely causes increased stability of the TOP2 cleavage complexes, which has been shown to result in a greater number of double-stranded breaks throughout the genome [28]. Therefore, we expect *C*. *elegans* strains that contain a methionine at this residue to accumulate more genomic damage when exposed to etoposide. Although we do not have direct data to support the cellular effect of the drug, we believe that increased genomic damage likely delays development and causes the progeny of exposed individuals to be shorter.

### TOP-2 variation causes allele-specific interactions with an expanded set of topoisomerase II poisons

**Fig 4:**
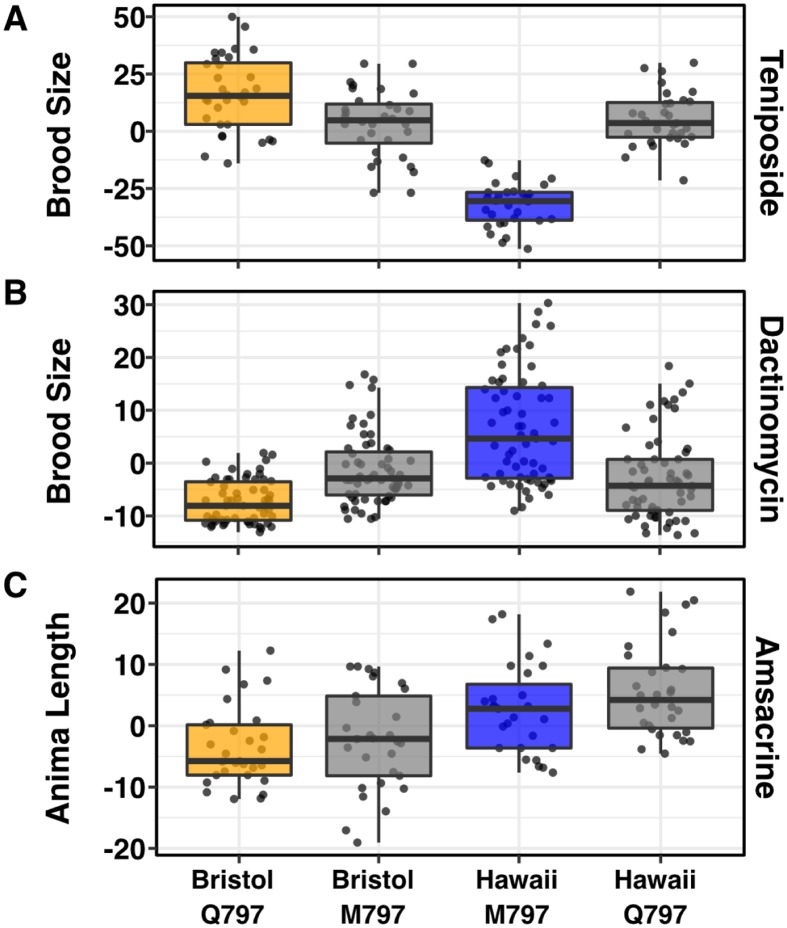
Variation in the TOP-2 797 allele underlies response to multiple topoisomerase II poisons. Tukey box plots of (A) regressed brood size in response to teniposide. Mean phenotypes of the Bristol and Hawaiian allele swap strains are significantly different from the Bristol and Hawaiin parental strains (Tukey’s HSD, Bristol—Bristol Swap *p-*value = 0.0009; Hawaii—Hawaiian Swap *p-*value < 1E-7). (B) Tukey box plots of regressed brood size in response to dactinomycin. Mean phenotypes of the Bristol and Hawaiian allele swap strains are significantly different from the Bristol and Hawaiin parental strains (Tukey’s HSD, Bristol—Bristol Swap *p-*value = 0.0002; Hawaii—Hawaiian Swap *p-*value = 3E-7). (C) Tukey box plots of regressed animal length in response to amsacrine show allele swap strains are not significantly different from parental strains (Tukey’s HSD, Bristol—Bristol Swap *p-*value = 0.925; Hawaii—Hawaiian Swap *p-*value = 0.414). Orange corresponds to the Bristol genetic background and blue to the Hawaii background. Labels correspond to the genetic background and the corresponding residue at position 797 of TOP-2 (Q for glutamine, M for methionine).

Because the molecular docking simulations explain the observed physiological effects of etoposide exposure, we hypothesized that the 797 residue of TOP-2 would mediate differential interactions with additional topoisomerase II poisons based on their chemical structures. Like etoposide, teniposide, dactinomycin, and amsacrine each contain core cyclic rings that are thought to interfere with the re-ligation step of the topoisomerase II catalytic cycle through DNA interactions [17]. However, the functional groups attached to the core cyclic rings of each poison vary in their polarity and size, which could affect interactions with topoisomerase II enzymes. For example, the only difference between teniposide and etoposide is the presence of a thienyl or methyl group attached to the D-glucose derivative, respectively, but they share a similarly sized and hydrophobic functional group. We predicted that these two drugs would have comparable interactions with the TOP-2 alleles and elicit a similar physiological response. By contrast, the polar functional groups of dactinomycin likely have stronger interactions with the glutamine variant and induce increased cytotoxicity in animals that contain this allele. We quantified the physiological responses of the TOP-2 allele-replacement strains exposed to these two drugs and found that each response matched our predictions (Fig 4). Specifically, the Bristol strain is more resistant to teniposide than the Hawaiian strain and the introduction of the M797 allele into the Bristol genetic background results in increased sensitivity. Similarly, the introduction of the Q797 TOP-2 allele into the Hawaiian genetic background resulted in increased teniposide resistance. Conversely, the Hawaiian strain is more resistant to dactinomycin treatment than the Bristol strain and the introduction of the Q797 allele into the Hawaiian genetic background results in increased sensitivity. Additionally, the introduction of the M797 TOP-2 allele into the Bristol genetic background resulted in increased dactinomycin resistance. In both of these cases the difference between the parental strain and the allele swap strain resulted in significant differences in the means (Tukey’s HSD). However, for both dactinomycin and teniposide the allele swap strains were not significantly different from each other. These results suggest that additional drug-specific QTL may exist for these two drugs. Unlike etoposide, teniposide, or dactinomycin, the core cyclic rings of amsacrine do not have an equivalent functional group to interact with the TOP-2 797 residue, suggesting that variation at TOP-2 residue 797 will have no impact on amsacrine sensitivity. Although the Bristol and Hawaiian strains differed, we found that the Bristol and Hawaiian allele swap strains had no quantifiable difference in amsacrine response when compared to the parental strains (Fig 4) and different genomic loci control response to this drug (S9 Fig). These results support the hypothesis that the polarity of the putative drug-binding pocket determines the cytotoxic effects of multiple, but not all, topoisomerase II poisons. To further explore this hypothesis, we tested a drug (XK469) that has preferential hTOPOIIβ specificity [29]. Surprisingly, we found that the Bristol allele swap strain was more sensitive to XK469 treatment than the parental Bristol strain, and the Hawaiian allele swap strain was more resistant to XK469 treatment than the parental Hawaiian strain (S10 Fig). This result indicates that an additional mechanism might contribute to XK469 specificity in human cells and underscores the importance of functional validation of specific residues that are thought to be involved in targeted drug binding.

### The equivalent site in topoisomerase II alpha causes differential susceptibility to diverse poisons in human cells

**Fig 5:**
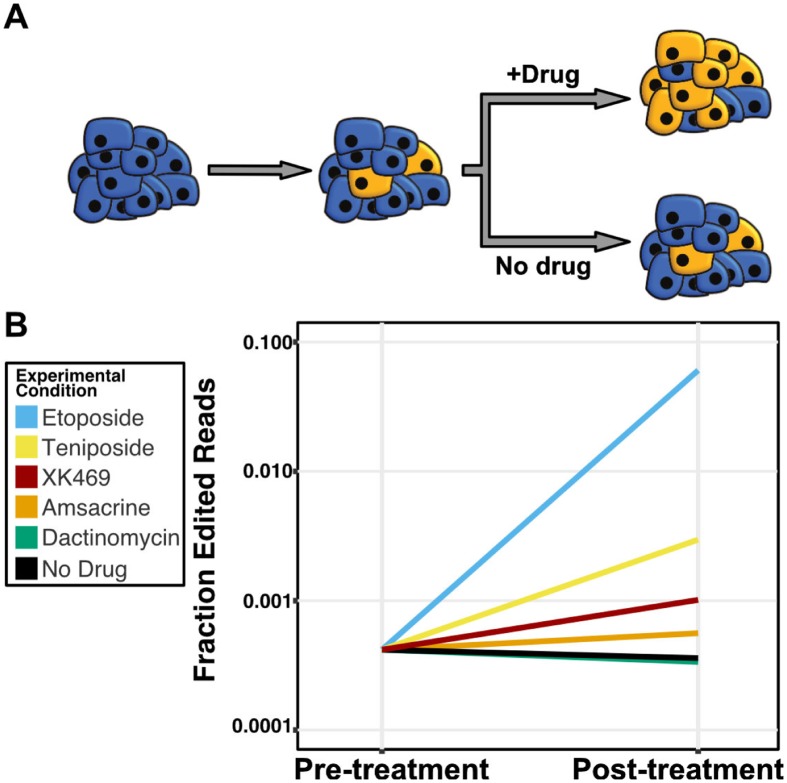
Human cells that contain hTOPOIIα M762Q are more resistant to etoposide, teniposide, and XK469. (A) Cartoon depiction of human cell-line experiment. A population of cells is incubated with CRISPR reagents to introduce the M762Q mutation in hTOPOIIα. A small fraction of cells are edited at this position and depicted in orange. Prior to splitting this population of cells into separate growth cultures with drug or control conditions, a subset of cells are prepared for sequencing to assess the fraction of edited cells. Split populations are then allowed to grow for two weeks and prepared for sequencing. In this example, blue cells contain the wt hTOPOIIα and the orange cells contain the edited M762Q hTOPOIIα, both strains contain the wt hTOPOIIβ. (B) Log-transformed fraction of sequencing reads that contain the CRISPR-edited allele of hTOPOIIα is plotted on the y axis for pre-treatment and post-treatment cell populations. The fraction of cells that contain the hTOPOIIα glutamine allele increase by 168.2-, 8.2-, 2.8-, and 1.6-fold upon treatment with etoposide, teniposide, XK469, and amsacrine, respectively, when compared to the no-drug control. All four of these enrichments of the edited glutamine allele were significant by Fisher’s exact test (etoposide and teniposide, *p*-value < 2.2E-16; amsacrine *p*-value = 0.025; XK469 *p*-value < 1.67E-13). The fraction of cells that contain the hTOPOIIα glutamine allele did not significantly increase in dactinomycin treated cells (Fisher’s exact test, *p*-value = 0.7182).

To determine if differences in the hydrophobicities of the two human topoisomerase II putative drug-binding pockets underlie etoposide sensitivity, we used CRISPR/Cas9 genome editing and a pooled-sequencing approach to create human embryonic kidney 293 cells (293T) that encode hTOPOIIα enzymes with a hTOPOIIβ-like drug-binding pocket. These two enzymes differ at the corresponding residue (M762 in hTOPIIα and Q778 in hTOPIIβ) that explains phenotypic variation in the natural *C*. *elegans* population exposed to etoposide. Human 293T cells were incubated with genome-editing machinery for six hours, allowed to recover for five days, and then split into two populations for etoposide exposure or no etoposide exposure. Etoposide treatment provided a selective pressure that upon further passaging led to a greater than 160-fold enrichment of cells that contain the glutamine-edited hTOPOIIα allele as compared to populations of cells exposed to no drug (Fig 5). These results show that cells with the glutamine-edited hTOPOIIα allele are more resistant to etoposide treatment than cells with the non-edited methionine hTOPOIIα allele. Notably, the rarity of genome editing events makes it unlikely that every copy of the hTOPOIIα gene in this diploid/polyploid cell line is edited. Because we see etoposide resistance in these incompletely edited cells, hTOPOIIα dimeric complexes likely contain one edited and one wild-type copy of hTOPOIIα and do not bind etoposide as well as causing less cytotoxicity. These data confirm both our dominance test (S6 Fig) and the two-drug model of etoposide binding [30] in which both enzymes of the homodimer must be bound by poison to be completely inhibited. Additionally, we performed the reciprocal experiment to edit the glutamine-encoding hTOPOIIβ gene to a version that encodes methionine. If the methionine hTOPOIIβ allele is more sensitive to etoposide than the glutamine hTOPOIIβ, we would expect to observe a depletion of methionine-edited cells upon etoposide treatment. However, because glutamine-to-methionine editing occurred in less than 1% of the cells, it was difficult to detect further reductions in methionine allele frequencies (S1 Table). Overall, we demonstrate that this residue underlies variation in etoposide response in the natural *C*. *elegans* population and human cell lines edited to contain the glutamine-edited hTOPOIIα enzyme.

Our *C*. *elegans* results using the genome-edited *top-2* strains show that variation at this residue underlies differences in some but not all topoisomerase II poisons. We exposed the edited human cell lines to these different poisons to test this hypothesis. We found that cells containing the glutamine-edited hTOPOIIα allele are less affected by both teniposide and XK469, as indicated by the respective 8.2- and 2.8-fold increase in edited cell frequency upon drug exposure as compared to cell populations with no drug added. These results mirror our observations that *C*. *elegans* strains with the methionine TOP-2 allele are more sensitive to these drugs. We observed moderate-to-no change in edited allele frequencies between cell populations exposed to amsacrine (~1.6-fold increase) or dactinomycin (~0.93-fold decrease) and those cells exposed to no-drug control conditions. These results indicate that the hTOPOIIα M762 residue does not interact with amsacrine. Our expectation was that dactinomycin would be more cytotoxic to cells that contain the glutamine hTOPOIIα allele, and therefore result in a depletion of edited cells. As mentioned above, we do not have the power to detect this depletion in response because of low CRISPR-editing efficiency. However, we did expect to see enrichment of hTOPOIIβ Q778M edited cells upon exposure to dactinomycin in the reciprocal experiment. Despite this expectation, we saw no change in hTOPOIIβ Q778M edited cell frequency between dactinomycin and the no drug control. Our inability to detect any enrichment of allele frequencies might result from dactinomycin having multiple cellular targets in addition to topoisomerase II [31]. Taken together, our findings testing a variety of poisons on human cell lines recapitulated the results from *C*. *elegans*.

## Discussion

Few genetic markers have been identified that predict patient responses to chemotherapeutic regimens [32,33]. The goal of this study was to introduce new methods for the rapid and cost-effective identification of genetic variants that explain differences in chemotherapeutic response. Our approach leveraged genetic and phenotypic variation present in the model organism *C*. *elegans* to identify a single amino acid variant (Q797M) in the topoisomerase II enzyme that underlies differences in etoposide response. Mechanistic insights into differential etoposide binding between the glutamine or methionine alleles gave us the power to predict the physiological responses to an expanded panel of topoisomerase II poisons. These results highlight how the combination of a highly sensitive phenotyping assay with classical and quantitative genetics approaches in *C*. *elegans* can rapidly identify the mechanistic underpinnings of phenotypic variability in response to a key class of antineoplastic compounds.

Our approach stands in stark contrast to previous underpowered human cell line [34] and clinical studies [35] that failed to identify any statistically significant associations between etoposide-induced cytotoxicity and genetic variation in the human population. However, the residue we identified in *C*. *elegans* does not vary in the human population [36], suggesting that GWA studies would not have identified this variant as a marker for etoposide sensitivity. Nevertheless, this residue is one of the few differences between the putative drug-binding pockets of the two human topoisomerase II isoforms (M762 in hTOPIIα and Q778 in hTOPIIβ), which allowed us to investigate the molecular underpinnings of drug binding. We verified that this single amino acid change in the human topoisomerase II isoforms results in profound differences in topoisomerase II poison-induced cytotoxicity using 293T cells. Though previous hTOPOIIβ structural studies have implicated this glutamine-methionine difference as functionally important for etoposide binding [21,22], studies involving hTOPOIIα have argued that this residue is not involved [23]. The results presented here unequivocally show that this residue contributes to differential topoisomerase II poison-induced cytotoxicity and have important implications for targeted drug design.

Although topoisomerase II poisons can bind and inhibit both hTOPOIIα and hTOPOIIβ, hTOPOIIα is the cellular target of poisons in most cancers because it is expressed in proliferating cells [17]. However, recent evidence suggests that side effects associated with these treatments are caused by inhibition of hTOPOIIβ in differentiated cells [37]. For example, antineoplastic treatment regimens that contain the epipodophyllotoxins (*e*.*g*. etoposide or teniposide) are hypothesized to increase the risk of developing secondary malignancies caused by hTOPOIIβ-dependent 11q23 translocations [38–41]. Additionally, the most severe side effects associated with treatments that contain an alternative class of topoisomerase II poisons (anthracyclines, *e*.*g*. doxorubicin or daunorubicin) include dose-dependent cardiotoxicity and heart failure dependent on hTOPOIIβ [42–44]. Therefore, optimal topoisomerase II poisons will maximize interactions with hTOPOIIα to inhibit proliferating cells and minimize hTOPOIIβ interactions to reduce side effects. With this goal in mind, others have identified etoposide analogues with different isoform specificities but have not determined the mechanism of specificity [45]. Our study functionally validates a key residue determining isoform specificity and is critical to the improvement of this widely administered drug class. The importance of such functional characterization is underscored by our observation that *C*. *elegans* strains and human cells with the methionine TOP-2 allele are more sensitive to XK469, despite this drug being shown to be a β-specific poison [29]. Though XK469 has been shown to be a β-specific poison, no information regarding its drug binding pocket or the mechanism driving isoform specificity is currently known. Our results indicate that XK469 occupies a similar drug-binding pocket of TOP-2 as other topoisomerase II poisons and interacts with residue 797.

To date, no human genetic variants have been linked to topoisomerase II poison-induced cytotoxicity. Of the 291 and 279 respective SNVs in hTOPOIIα and hTOPOIIβ that encode missense mutations (S14 and S15 Tables) found in the ExAC database [36], some are near the highly conserved DNA-binding domains or drug-binding pockets (S11 Fig), which could affect drug response. However, the extent to which these variants impact responses to topoisomerase II poisons is unknown, so functional validation is required. The approach of editing human cells and following allele frequencies via sequencing represents a scalable method to assess the functional role of these variants and avoids single-cell cloning. Importantly, differences in responses to topoisomerase II poisons might not be affected by variation in the topoisomerase II isoforms but instead mediated by variation in cellular import, metabolism, or export. Pharmacogenomic data available for many antineoplastic compounds [46,47], in combination with human variation data [36], can be used to prioritize and test variants in highly conserved regions of proteins known to be involved in these alternative processes. This biased approach focused on candidate variants is necessitated by the lack of power in clinical GWA studies and is not guaranteed to successfully connect variants to differences in drug response. For this reason, unbiased mapping approaches in model organisms combined with functional validation in genome-edited human cells will greatly expand our current understanding of how human genetic variation affects drug responses.

## Materials and methods

### Strains

Animals were cultured at 20°C with the bacterial strain OP50 on modified nematode growth medium (NGM), containing 1% agar and 0.7% agarose to prevent burrowing of the wild isolates. For each assay, strains were grown at least five generations with no strain entering starvation or encountering dauer-inducing conditions [48]. Wild *C*. *elegans* isolates used for genome-wide association are described previously [24,49]. Recombinant inbred advanced intercross lines (RIAILs) used for linkage mapping were constructed previously [16]. Strains constructed for this manuscript are listed in Supplemental Information. Construction of individual strains is detailed in the corresponding sections below.

### High-throughput fitness assay

We used a modified version (S1 Fig) of the high-throughput fitness assay (HTA) described previously [16]. In short, strains are passaged for four generations to reduce transgenerational effects from starvation or other stresses. Strains are then bleach-synchronized and aliquoted to 96-well microtiter plates at approximately one embryo per microliter in K medium [50]. Embryos are then hatched overnight to the L1 larval stage. The following day, hatched L1 animals are fed HB101 bacterial lysate (Pennsylvania State University Shared Fermentation Facility, State College, PA) at a final concentration of 5 mg/ml and grown to the L4 stage after two days at 20°C. Three L4 larvae are then sorted using a large-particle flow cytometer (COPAS BIOSORT, Union Biometrica, Holliston, MA) into microtiter plates that contain HB101 lysate at 10 mg/ml, K medium, 31.25 μM kanamycin, and either drug dissolved in 1% DMSO or 1% DMSO. The animals are then grown for four days at 20°C. During this time, the animals will mature to adulthood and lay embryos that encompass the next generation. Prior to the measurement of fitness parameters from the population, animals are treated with sodium azide (50 mM) to straighten their bodies for more accurate length measurements. Traits that are measured by the BIOSORT include brood size and animal length (time of flight or TOF).

### Calculation of fitness traits for genetic mappings

Phenotype data generated using the BIOSORT were processed using the R package *easysorter*, which was specifically developed for processing this type of data set [51]. Briefly, the function *read_data*, reads in raw phenotype data, runs a support vector machine to identify and eliminate bubbles. Next, the *remove_contamination* function eliminates any wells that were contaminated prior to scoring population parameters for further analysis. Contamination is assessed by visual inspection. The *sumplate* function is then used to generate summary statistics of the measured parameters for each animal in each well. These summary statistics include the 10th, 25th, 50th, 75th, and 90th quantiles for TOF. Measured brood sizes are normalized by the number of animals that were originally sorted into the well. After summary statistics for each well are calculated, the *regress(assay = TRUE)* function in the *easysorter* package is used to fit a linear model with the formula (*phenotype ~ assay*) to account for any differences between assays. Next, outliers are eliminated using the *bamf_prune* function. This function eliminates strain values that are greater than two times the IQR plus the 75th quantile or two times the IQR minus the 25th quantile, unless at least 5% of the strains lie outside this range. Finally, drug-specific effects are calculated using the *regress(assay = FALSE)* function from *easysorter*, which fits a linear model with the formula (*phenotype ~ control phenotype*) to account for any differences in population parameters present in control DMSO-only conditions.

### Topoisomerase II poisons dose-response assays

All dose-response experiments were performed on four genetically diverged strains (Bristol, Hawaii, DL238, and JU258) in technical quadruplicates prior to performing GWA and linkage mapping experiments (S4 Table). Animals were assayed using the HTA, and phenotypic analysis was performed as described above. Drug concentrations for GWA and linkage mapping experiments were chosen based on two criteria—an observable drug-specific effect and broad-sense heritability *H*^2^. We aimed to use the first concentration for which a drug-specific effect with a maximum *H*^2^ was observed. Broad-sense heritability estimates were calculated using the *lmer* function in the *lme4* package with the following model (*phenotype ~1 + (1|strain)*). Concentrations for each chemotherapeutic used in mapping experiments are; etoposide—250 μM, teniposide—125 μM, amsacrine—50 μM, dactinomycin—15 μM, and XK469–1000 μM. All topoisomerase II poisons used in this study were purchased from Sigma (XK469 cat#X3628, etoposide cat#E1383, amsacrine cat#A9809, dactinomycin cat#A1410, and teniposide cat#SML0609).

### Linkage mapping

A total of 265 RIAILs were phenotyped in the HTA described previously for control and etoposide conditions. The phenotype data and genotype data were entered into R and scaled to have a mean of zero and a variance of one for linkage analysis (S5 Table). Quantitative trait loci (QTL) were detected by calculating logarithm of odds (LOD) scores for each marker and each trait as −*n*(*ln*(1−*r*^2^)/2*ln*(10)), where *r* is the Pearson correlation coefficient between RIAIL genotypes at the marker and phenotype trait values [11]. The maximum LOD score for each chromosome for each trait was retained from three iterations of linkage mappings (S6 Table). We randomly permuted the phenotype values of each RIAIL while maintaining correlation structure among phenotypes 1000 times to estimate significance empirically. The ratio of expected peaks to observed peaks was calculated to determine the genome-wide error rate of 5% of LOD 4.61. Broad-sense heritability was calculated as the fraction of phenotypic variance explained by strain from fit of a linear mixed-model of repeat phenotypic measures of the parents and RIAILs [52]. The total variance explained by each QTL was divided by the broad-sense heritability to determine how much of the heritability is explained by each QTL. Confidence intervals were defined as the regions contained within a 1.5 LOD drop from the maximum LOD score.

### Genome-wide association mapping

Genome-wide association (GWA) mapping was performed using phenotype data of 152 *C*. *elegans* isotypes (S4 Table). We used the *cegwas* R package for association mapping [49]. This package uses the EMMA algorithm for performing association mapping and correcting for population structure [53], which is implemented by the GWAS function in the *rrBLUP* package [54]. Specifically, the *GWAS* function in the *rrBLUP* package was called with the following command: rrBLUP::GWAS(pheno = ph, geno = y, K = kin, min.MAF = 0.05, n.core = 1, P3D = FALSE, plot = FALSE). The kinship matrix used for association mapping was generated using a whole-genome high-quality single-nucleotide variant (SNV) set [24] and the *A*.*mat* function from the *rrBLUP* package. SNVs previously identified using RAD-seq [55] that had at least 5% minor allele frequency in the 152 isotype set were used for performing GWA mappings. Association mappings that contained at least one SNV that had a value greater than the Bonferroni-corrected value were processed further using fine mapping (S5 Table). Tajima’s D was calculated using the *tajimas_d* function in the *cegwas* package using default parameters (window size = 300 SNVs, sliding window distance = 100 SNVs, outgroup = N2) (S7 Table).

### Fine mapping

Fine mapping was performed on variants from the whole-genome high-quality SNV set within a defined region of interest for all mappings that contained a significant QTL. Regions of interest surrounding a significant association were determined by simulating a QTL with 20% variance explained at every RAD-seq SNV present in 5% of the phenotyped population. We then identified the most correlated SNV for each mapping. Next, we determined the number of SNVs away from the simulated QTL SNV position that captured 95% of the most correlated SNVs. A range of 50 SNVs upstream or downstream of the peak marker captured 95% of the most significant SNVs in the simulated mappings. We therefore used a region 50 SNVs from the last SNV above the Bonferroni-corrected *p*-value on the left side of the peak marker and 50 SNVs from the last SNV above the Bonferroni-corrected *p*-value on the right side of the peak marker.

The *snpeff* function from the *cegwas* package was used to identify SNVs from the whole-genome SNV set with high to moderate predicted functional effects present in a given region of interest [56]. The *p*-value associated with the Spearman’s correlation coefficient between each variant in the region of interest and the kinship-corrected phenotype used in the GWA mapping was calculated using the *variant_correlation* function and processed using the *process_correlations* function in the *cegwas* package (S10 Table). These functions use the *cor*.*test* function in R to calculate the *p*-value. ClustalX was used to perform the multiple sequence alignment between various topoisomerase II orthologs (S1 File).

### Near-isogenic line generation

NILs were generated by crossing N2xCB4856 RIAILs to each parental genotype. For each NIL, eight crosses were performed followed by six generations of selfing to homozygose the genome. Reagents used to generate NILs are detailed in Supplemental Information. The NILs responses to 250 μM etoposide were quantified using the HTA fitness assay described above (S8 Table).

### Dominance tests

Dominance experiments were performed using the fluorescent reporter strain EG7952 *oxTi207 [eft-3p*::*GFP*::*unc-54 3'UTR + hsp*::*peel-1 + NeoR + Cbr-unc-119(+)]*. Hermaphrodites of N2 and CB4856 were crossed to male EG7952 reporter strain, which expresses GFP, to ensure that we could measure heterozygous cross progeny by the presence of GFP. Three GFP-positive progeny were manually transferred to a 96-well assay microtiter plate containing to 250 μM etoposide dissolved in 1% DMSO or 1% DMSO control, in addition to K medium, HB101 lysate at 10 mg/ml, and 31.25 μM kanamycin. Animals were grown for four days at 20°C. The phenotypes of the progeny were scored using the BIOSORT as described above (S9 Table). Heterozygous progeny were computationally identified as those individuals that had fluorescence levels between the non-fluorescent and fluorescent parental strains.

### *top-2* and *npp-3* complementation

To perform the complementation experiments, N2 and CB4856 males were both crossed to both VC1474 *top-2(ok1930)/mIn1 [mIs14 dpy-10(e128)]* and VC1505 *npp-3(ok1900)/mIn1 [mIs14 dpy-10(e128)]* hermaphrodites. Three non-GFP L4 hermaphrodite progeny were manually picked into experimental wells containing either 250 μM etoposide dissolved in 1% DMSO or 1% DMSO without etoposide, in addition to HB101 lysate at 10 mg/ml, K medium, and 31.25 μM kanamycin. Animals were grown for four days at 20°C. The phenotypes of the progeny were scored using the BIOSORT as described above (S10 Table).

### *top-2* reciprocal hemizygosity

VC1474 *top-2(ok1930)/mIn1 [mIs14 dpy-10(e128)]* was used for *top-2* complementation tests. *top-2(ok1930)* and *mIn1[mIs14 dpy-10(e128)]* were individually introgressed into CB4856 for 10 generations. Once individual crosses were completed, CB4856 *mIn1 [mIs14 dpy-10(e128)]* was crossed to CB4856 *top-2(ok1930)* to generate ECA338, which contains a *mIn1*-balanced *top-2(ok1930)*. oECA1003 and oECA1004 were used to verify the presence of *top-2(ok1930)* during crosses.

To perform the reciprocal hemizygosity experiment, N2 and CB4856 males were both crossed to both VC1474 and ECA338 hermaphrodites. Three non-GFP L4 hermaphrodite progeny were manually picked into experimental wells containing either 250 μM etoposide dissolved in 1% DMSO or 1% DMSO without etoposide, in addition to HB101 lysate at 10 mg/ml, K medium, and 31.25 μM kanamycin. Animals were grown for four days at 20°C. The phenotypes of the progeny were scored using the BIOSORT as described above (S11 Table).

### Generation of *top-2* allele replacement strains

All allele replacement strains were generated using CRISPR/Cas9-mediated genome engineering, using the co-CRISPR approach [57] with Cas9 ribonucleoprotein delivery [58]. Alt-R^™^ crRNA and tracrRNA (Supplemental Information) were purchased from IDT (Skokie, IL). tracrRNA (IDT, 1072532) was injected at a concentration of 13.6 μM. The *dpy-10* and the *top-2* crRNAs were injected at 4 μM and 9.6 μM, respectively. The *dpy-10* and the *top-2* single-stranded oligodeoxynucleotides (ssODN) repair templates were injected at 1.34 μM and 4 μM, respectively. Cas9 protein (IDT, 1074182) was injected at 23 μM. To generate injection mixes, the tracrRNA and crRNAs were incubated at 95°C for 5 minutes and 10°C for 10 minutes. Next, Cas9 protein was added and incubated for 5 minutes at room temperature. Finally, repair templates and nuclease-free water were added to the mixtures and loaded into pulled injection needles (1B100F-4, World Precision Instruments, Sarasota, FL). Individual injected *P*_*0*_ animals were transferred to new 6 cm NGM plates approximately 18 hours after injections. Individual *F*_*1*_ rollers were then transferred to new 6 cm plates and allowed to generate progeny. The region surrounding the desired Q797M (or M797Q) edit was then amplified from *F*_*1*_ rollers using oECA1087 and oECA1124. The PCR products were digested using the *Hpy*CH4III restriction enzyme (R0618L, New England Biolabs, Ipswich, MA). Differential band patterns signified successfully edited strains because the N2 Q797, which is encoded by the CAG codon, creates an additional *Hpy*CH4III cut site. Non-Dpy, non-Rol progeny from homozygous edited *F*_*1*_ animals were propagated. If no homozygous edits were obtained, heterozygous *F*_*1*_ progeny were propagated and screened for presence of the homozygous edits. *F*_*1*_ and *F*_*2*_ progeny were then Sanger sequenced to verify the presence of the proper edit. Allele swap strains responses to the topoisomerase II poisons were quantified using the HTA fitness assay described above (S12 Table).

### Statistical analyses

All *p*-values testing the differences of strain phenotypes in the NIL, complementation, and allele-replacement experiments were performed in R using the *TukeyHSD* function on an anova model with the formula (*phenotype ~ strain*). *p*-values of individual pairwise strain comparisons were reported.

### Molecular docking simulations

The *C*. *elegans* TOP-2 three-dimensional structure homology model was built by threading the *C*. *elegans* TOP-2 peptide to the human topoisomerase II beta structure (PDB accession code 3QX3; 59% identity, 77% similarity) using the Prime3.1 module implemented in Schrodinger software [59,60]. After building the model, a robust energy minimization was carried out in the Optimized Potentials for Liquid Simulations (OPLS) force field. The minimized structure was subjected to MolProbity analysis, and the MolProbity score suggested with greater than 95% confidence that the minimized structure model was a good high-resolution structure [61].

Next, the Prot-Prep wizard was used to prepare the TOP-2 homology model, which fixed the hydrogen in the hydrogen bond orientations, eliminated the irrelevant torsions, fixed the missing atoms, assigned the appropriate force field charges to the atoms [62]. After preparing the structure, the glutamine 797 was mutated to various rotamers of methionine (Q797M), which subsequently underwent minimization in the OPLS force field. The energy-minimized structure was used in the *in silico* experiments.

The structure data file of etoposide (DrugBank ID: DB00773) was obtained from PubMed and was subjected to ligand preparation panel of the Schrodinger software. Using the induced fit docking (IFD) module of Schrodinger and Suflex software, we carried out the docking of etoposide with the glutamine (S2 File) and methionine (S3 File) forms of the *C*. *elegans* TOP-2 homology model. After the docking experiments, we analyzed the docked poses of the ligands bound to the TOP-2 homology models from both docking engines. Change in free energy (Δ*G*) and the hydrophobicity parameter were calculated using Schrodinger.

### CRISPR-Cas9 gene editing in human cells

Gene-editing experiments were performed in a single parallel culture experiment using human 293T cells (ATCC) grown in DMEM with 10% FBS. On day zero, 500,000 cells were seeded per well in a six-well plate format. The following day, two master mixes were prepared: a) LT-1 transfection reagent (Mirus) was diluted 1:10 in Opti-MEM and incubated for five minutes; b) a DNA mix of 500 ng Cas9-sgRNA plasmid with 250 pmol repair template oligonucleotide (Supplemental Information) was diluted in Opti-MEM in a final volume of 100 μL. 100 μL of the lipid mix was added to each of the DNA mixes and incubated at room temperature for 25 minutes. Following incubation, the full 200 μL volume of DNA and lipid mix was added drop-wise to the cells, and the cells were centrifuged at 1000xg for 30 min. Six hours post-transfection, the media on the cells was replaced, and the cells were passaged as needed. On day six, five million cells from each condition were pelleted to serve as an early time point for the editing efficiency, and five million cells were then passaged on the five drugs at two doses for 12 days, at which time all surviving cells were pelleted. Concentrations used for each small molecule are: etoposide– 500 nM, 100 nM; amasacrine– 500 nM, 100 nM; teniposide– 20 nM, 4 nM; dactinomycin– 4 nM, 800 pM; and XK469–5 μM, 1 μM.

### Analysis of CRISPR-Cas9 editing in human cells

Genomic DNA was extracted from cell pellets using the QIAGEN (QIAGEN, Hilden, Germany) Midi or Mini Kits based on the size of the cell pellet (cat # 51183, 51104) according to the manufacturer’s recommendations. TOP2A and B loci were first amplified with 17 cycles of PCR using a touchdown protocol and the NEBnext 2x master mix (New England Biolabs M0541). The resulting product served as input to a second PCR, using primers that appended a sample-specific barcode and the necessary adaptors for Illumina sequencing. The resulting DNA was pooled, purified with SPRI beads (A63880, Beckman Coulter, Brea, CA), and sequenced on an Illumina MiSeq with a 300 nucleotide single-end read with an eight nucleotide index read. For each sample, the number of reads exactly matching the wild-type and edited TOP2A/B sequence were determined (S13 Table). The numbers of reads were compared using a Fisher’s exact test.

## Supporting information

S1 Fig High-throughput fitness assay. Individual strains are passaged for four generations on agar plates seeded with OP50 bacteria by transfer of seven L4 larval stage animals to a fresh plate each generation every three days. Animals are bleach synchronized and aliquoted to 96-well microtiter plates in 50 μl of K medium at a concentration of one embryo per μL. Aliquoted embryos are incubated overnight at 20°C. The following day, 5 μl of 50 mg/ml HB101 lysate is added to each well. Animals are then grown for two days to the L4 larval stage. Then, three L4 animals are sorted to assay plates containing drug or DMSO using the BIOSORT. Four days later, 200 μl M9 plus 50 mM sodium azide is added to each well and strains are scored using the BIOSORT.
(TIFF)

S2 Fig Dose response curves of four genetically diverged *C*. *elegans* strains. For each drug, concentration in μM is plotted on the x-axis, and the regressed 75th quantile of animal length is plotted on the y-axis. The red numbers above the dose response lines correspond to broad-sense heritability estimates. The heritability estimates for the drug concentrations used in experiments throughout this study are: *H*^*2*^ = 0.47 for amsacrine at 50 μM, *H*^*2*^ = 0.62 for etoposide at 250 μM, *H*^*2*^ = 0.73 for teniposide at 125 μM, and *H*^*2*^ = 0.9 for XK469 at 1000 μM. The Bristol strain (N2) is colored in orange, the Hawaiian strain (CB4856) in blue, JU258 in cyan, and DL238 in green.
(TIFF)

S3 Fig Near isogenic lines animal length upon exposure to etoposide. Tukey box plots of NIL regressed median animal length in the presence of etoposide are shown. NIL genotypes are indicated below the plot by colored rectangles, Bristol (orange) or Hawaii (blue). Numbers above rectangles correspond to the genomic position of the introgression region on chromosome II.
(TIFF)

S4 Fig Tajima’s D across the etoposide QTL confidence interval. Divergence, as measured by Tajima's D, is shown across the etoposide QTL confidence interval (II:11021073–12008179). The whole-genome SNV data set [24,49] was used for Tajima’s D calculations. Window size for the calculations was 300 SNVs with a 100 SNV sliding window size. The vertical red line marks the position of the *top-2* locus. The *tajimas_d* function in the *cegwas* package was used to perform the calculations.
(TIFF)

S5 Fig The worldwide distribution of the TOP-2(Q797M) allele. Glutamine (REF) is shown in orange and methionine (ALT) is shown in blue. Latitude and longitude coordinates of sampling locations were used to plot individual strains on the map [49].
(TIFF)

S6 Fig Dominance test for etoposide sensitivity. Tukey box plots of Bristol and Hawaii regressed median animal length in response to etoposide are plotted in orange and blue, respectively. GFP-containing Bristol strains (EG7952) were crossed to the Bristol and Hawaii strains and heterozygous progeny were assayed using the high-throughput fitness assay. Tukey box plots of heterozygotes are shown in gray. All heterozygote strains are significantly different from the parental Bristol and Hawaii strains (Tukey’s HSD, *p* < 0.02). All heterozygote strains are not significantly different from each other (Tukey’s HSD, *p* > 0.91).
(TIFF)

S7 Fig Complementation tests for *top-2* and *npp-3*. Tukey box plots of the residual brood size distribution of Bristol (orange) and Hawaii (blue) compared to two heterozygous (A) *top-2* and (B) *npp-3* deletion strains (gray) in response to etoposide are plotted. Orange (Bristol) and blue (Hawaii) rectangles below the plot correspond to the two chromosome II homolog genotypes. Gray triangles denote chromosomes with the *top-2* deletion allele. (A) The Bristol strain and the Bristol/Bristol(Δ*top-2*) heterozygous strain are not significantly different from each other (Tukey’s HSD, *p*-value 0.104). The Hawaii strain and the Hawaii/Bristol(Δ*top-2*) heterozygous strain are not significantly different from each other (Tukey’s HSD, *p*-value 0.226). All other comparisons are significant (Tukey’s HSD *p*-value < 4.1E-6). (B) The Hawaii/Bristol(Δ*npp-3*) and the Bristol/Bristol(Δ*npp-3*) heterozygous strain are not significantly different from each other (Tukey’s HSD, *p*-value 0.68), but all other comparisons are significant (Tukey’s HSD *p*-value < 0.009).
(TIFF)

S8 Fig Complete *top-2* reciprocal hemizygosity experiment. (A) Tukey box plots of the residual median animal length distribution of the Bristol (orange) and Hawaii (blue) strains compared to four heterozygous *top-2* deletion strains (gray) in response to etoposide are plotted. Orange (Bristol) and blue (Hawaii) rectangles below the plot correspond to the two chromosome II homolog genotypes. Gray triangles denote chromosomes with the *top-2* deletion allele. Phenotypes of heterozygous deletion strains with the Bristol TOP-2 allele are not significantly different from the parental Bristol strain. The Hawaii/Bristol(Δ*top-2*) and the Hawaii/Hawaii(Δ*top-2*) strain phenotypes are not significantly different from each other (Tukey’s HSD, *p-*value = 0.16), but are both significantly different from the parental Hawaii strain (Tukey’s HSD, *p-*value < 1E-7 and *p*-value = 0.0001, respectively).
(TIFF)

S9 Fig GWA and linkage mapping amsacrine response. (A) A manhattan plot for regressed fraction of animals in the L1 larval stage in the presence of amsacrine is plotted. Each dot represents an SNV that is present in at least 5% of the phenotyped population. The –*log*_10_(*p*) for each SNV is plotted on the y-axis and the genomic position (Mb) is on the x-axis. Each tick on the x-axis corresponds to 5 Mb. SNVs are colored red if they pass the genome-wide Bonferroni-corrected significance threshold, which is denoted by the gray horizontal line. The genomic regions of interest are represented by cyan rectangles surrounding each QTL. (B) A linkage mapping plot for regressed fraction of animals in the L1 larval stage in the presence of amsacrine is shown. The significance value (logarithm of odds, LOD, ratio) is plotted on the y-axis and genomic position (Mb), separated by chromosome, on the x-axis. Each tick on the x-axis corresponds to 5 Mb. The associated 1.5 LOD-drop confidence intervals are represented by blue bars.
(TIFF)

S10 Fig Phenotypic responses to XK469 of allele-replacement strains. Tukey box plots of regressed median animal length in response to XK469 are plotted. Orange corresponds to the Bristol genetic background and blue to Hawaii. Allele-replacement strains are denoted by their genetic background and the corresponding residue change at position 797 of TOP-2. Strains containing the glutamine TOP-2 allele are more resistant to XK469 than strains with the methionine allele. All strain comparisons are significantly different from each other (Tukey’s HSD, *p*-value < 0.01)
(TIFF)

S11 Fig Variant residues in human topoisomerase II enzyme isoforms. (A) hTOPOIIα (PDB 4FM9) and (B) hTOPIIβ (PDB 3QX3) are shown. Putative etoposide-binding residues are highlighted in red, DNA-binding residues are highlighted in yellow, and the catalytic tyrosine is highlighted in orange. The cartoon representation of the peptide backbones is shown in tan, and DNA is shown in gray. Residues that vary in the human population are highlighted in purple.
(TIFF)

S1 Table Dose response phenotype data. Processed dose-response phenotype data used for establishing the doses to use for mapping experiments.
(CSV)

S2 Table RIAIL phenotype data. Processed phenotype data of RIAILs in the presence of amsacrine or etoposide used to perform linkage mapping.
(CSV)

S3 Table Linkage mapping LOD scores. Processed linkage mapping LOD scores at markers across the genome for median animal length in the presence of etoposide.
(CSV)

S4 Table Wild isolate phenotype data. Processed phenotype data of wild isolates in the presence of amsacrine or etoposide used to perform GWA mapping.
(CSV)

S5 Table GWA mapping significance values. Processed GWA mapping significance values for markers across the genome in the presence of amsacrine of etoposide.
(CSV)

S6 Table Variant gene correlation values. Spearman’s *rho* *p*-values for all coding variants present in the etoposide median animal length confidence interval.
(CSV)

S7 Table Tajima’s D values. Sliding window Tajimas’s D values for the etoposide median animal length confidence interval.
(CSV)

S8 Table NIL phenotype data. Processed median animal length phenotype data of NILs in the presence of etoposide.
(CSV)

S9 Table Dominance test phenotype data. Processed median animal length phenotype data of strains used to perform the dominance test in the presence of etoposide.
(CSV)

S10 Table *npp-3* and *top-2* complementation test phenotype data. Processed brood size phenotype data of strains used to perform the *npp-3* and *top-2* complementation tests in the presence of etoposide.
(CSV)

S11 Table *top-2* reciprocal hemizygosity test phenotype data. Processed median animal length phenotype data of strains used to perform the *top-2* reciprocal hemizygosity test in the presence of etoposide.
(CSV)

S12 Table CRISPR allele-swap strains phenotype data. Processed phenotype data of CRISPR allele-swap strains in the presence of etoposide.
(CSV)

S13 Table hTOP2A and hTOP2B read counts. Processed read counts of hTOP2A and hTOP2B prior to and after exposure to amsacrine, dactinomycin, etoposide, teniposide, XK469, or no drug.
(CSV)

S14 Table hTOP2A variants. Natural variants present in human *TOP2A*.
(CSV)

S15 Table hTOP2B variants. Natural variants present in human *TOP2B*.
(CSV)

S1 File TOP2 multiple sequence alignment. Multiple sequence alignment of multiple TOP2 orthologues.
(ALN)

S2 File *C*. *elegans* TOP-2(Q797) homology model. *C*. *elegans* TOP-2(Q797) 3-dimensional homology model.
(PDB)

S3 File *C*. *elegans* TOP-2(M797) homology model. *C*. *elegans* TOP-2(M797) 3-dimensional homology model.
(PDB)

S1 Supplemental Methods Supplemental methods are attached in a separate document. (DOCX)

## References

[1] Visscher PM, Brown MA, McCarthy MI, Yang J. Five years of GWAS discovery. Am J Hum Genet. 2012;90: 7–24. doi: 10.1016/j.ajhg.2011.11.029 22243964 10.1016/j.ajhg.2011.11.029 PMC3257326

[2] Boddy AV. Genetics of cisplatin ototoxicity: confirming the unexplained? Clin Pharmacol Ther. 2013;94: 198–200. doi: 10.1038/clpt.2013.116 23872836 10.1038/clpt.2013.116

[3] Liu J, Huang J, Zhang Y, Lan Q, Rothman N, Zheng T, et al Identification of gene-environment interactions in cancer studies using penalization. Genomics. 2013;102: 189–194. doi: 10.1016/j.ygeno.2013.08.006 23994599 10.1016/j.ygeno.2013.08.006 PMC3869641

[4] Hunter DJ. Gene-environment interactions in human diseases. Nat Rev Genet. 2005;6: 287–298. doi: 10.1038/nrg1578 15803198 10.1038/nrg1578

[5] Koboldt DC, Steinberg KM, Larson DE, Wilson RK, Mardis ER. The next-generation sequencing revolution and its impact on genomics. Cell. 2013;155: 27–38. doi: 10.1016/j.cell.2013.09.006 24074859 10.1016/j.cell.2013.09.006 PMC3969849

[6] Park J-H, Gail MH, Greene MH, Chatterjee N. Potential usefulness of single nucleotide polymorphisms to identify persons at high cancer risk: an evaluation of seven common cancers. J Clin Oncol. American Society of Clinical Oncology; 2012;30: 2157–2162. doi: 10.1200/JCO.2011.40.1943 22585702 10.1200/JCO.2011.40.1943 PMC3397697

[7] Willoughby LF, Schlosser T, Manning SA, Parisot JP, Street IP, Richardson HE, et al An in vivo large-scale chemical screening platform using Drosophila for anti-cancer drug discovery. Dis Model Mech. 2013;6: 521–529. doi: 10.1242/dmm.009985 22996645 10.1242/dmm.009985 PMC3597034

[8] Perlstein EO, Ruderfer DM, Roberts DC, Schreiber SL, Kruglyak L. Genetic basis of individual differences in the response to small-molecule drugs in yeast. Nat Genet. 2007;39: 496–502. doi: 10.1038/ng1991 17334364 10.1038/ng1991

[9] King EG, Kislukhin G, Walters KN, Long AD. Using Drosophila melanogaster to identify chemotherapy toxicity genes. Genetics. 2014;198: 31–43. doi: 10.1534/genetics.114.161968 25236447 10.1534/genetics.114.161968 PMC4174942

[10] Ehrenreich IM, Torabi N, Jia Y, Kent J, Martis S, Shapiro JA, et al Dissection of genetically complex traits with extremely large pools of yeast segregants. Nature. 2010;464: 1039–1042. doi: 10.1038/nature08923 20393561 10.1038/nature08923 PMC2862354

[11] Bloom JS, Ehrenreich IM, Loo WT, Lite T-LV, Kruglyak L. Finding the sources of missing heritability in a yeast cross. Nature. Nature Publishing Group; 2013;494: 1–6. doi: 10.1038/nature11867 23376951 10.1038/nature11867 PMC4001867

[12] Demogines A, Smith E, Kruglyak L, Alani E. Identification and dissection of a complex DNA repair sensitivity phenotype in Baker’s yeast. PLoS Genet. 2008;4: e1000123 doi: 10.1371/journal.pgen.1000123 18617998 10.1371/journal.pgen.1000123 PMC2440805

[13] Liti G, Louis EJ. Advances in quantitative trait analysis in yeast. PLoS Genet. 2012;8: e1002912 doi: 10.1371/journal.pgen.1002912 22916041 10.1371/journal.pgen.1002912 PMC3420948

[14] Stern DL. Identification of loci that cause phenotypic variation in diverse species with the reciprocal hemizygosity test. Trends Genet. 2014;30: 547–554. doi: 10.1016/j.tig.2014.09.006 25278102 10.1016/j.tig.2014.09.006

[15] Ghosh R, Andersen EC, Shapiro JA, Gerke JP, Kruglyak L. Natural Variation in a Chloride Channel Subunit Confers Avermectin Resistance in C. elegans. Science. 2012;335: 574–578. doi: 10.1126/science.1214318 22301316 10.1126/science.1214318 PMC3273849

[16] Andersen EC, Shimko TC, Crissman JR, Ghosh R, Bloom JS, Seidel HS, et al A Powerful New Quantitative Genetics Platform, Combining *Caenorhabditis elegans* High-Throughput Fitness Assays with a Large Collection of Recombinant Strains. G3. Genetics Society of America; 2015;5: g3.115.017178–920. doi: 10.1534/g3.115.017178 25770127 10.1534/g3.115.017178 PMC4426375

[17] Pommier Y, Leo E, Zhang H, Marchand C. DNA Topoisomerases and Their Poisoning by Anticancer and Antibacterial Drugs. Chem Biol. Elsevier Ltd; 2010;17: 421–433. doi: 10.1016/j.chembiol.2010.04.012 20534341 10.1016/j.chembiol.2010.04.012 PMC7316379

[18] Pommier Y, Schwartz RE, Kohn KW, Zwelling LA. Formation and rejoining of deoxyribonucleic acid double-strand breaks induced in isolated cell nuclei by antineoplastic intercalating agents. Biochemistry. 1984;23: 3194–3201. Available: https://www.ncbi.nlm.nih.gov/pubmed/6087890 6087890 10.1021/bi00309a013

[19] Gómez-Herreros F, Romero-Granados R, Zeng Z, Álvarez-Quilón A, Quintero C, Ju L, et al TDP2-dependent non-homologous end-joining protects against topoisomerase II-induced DNA breaks and genome instability in cells and in vivo. PLoS Genet. 2013;9: e1003226 doi: 10.1371/journal.pgen.1003226 23505375 10.1371/journal.pgen.1003226 PMC3592926

[20] Nitiss JL. Targeting DNA topoisomerase II in cancer chemotherapy. Nat Rev Cancer. 2009;9: 338–350. doi: 10.1038/nrc2607 19377506 10.1038/nrc2607 PMC2748742

[21] Wu C-C, Li T-K, Farth L, Lin L-Y, Lin T-S, Yu Y-J, et al Structural Basis of Type II Topoisomerase Inhibition by the Anticancer Drug Etoposide. Science. 2011;333: 456–459. 21778401 10.1126/science.1204117

[22] Wu C-C, Li Y-C, Wang Y-R, Li T-K, Chan N-L. On the structural basis and design guidelines for type II topoisomerase-targeting anticancer drugs. Nucleic Acids Res. Oxford University Press; 2013;41: 10630–10640. doi: 10.1093/nar/gkt828 24038465 10.1093/nar/gkt828 PMC3905874

[23] Wendorff TJ, Schmidt BH, Heslop P, Austin CA, Berger JM. The structure of DNA-bound human topoisomerase II alpha: conformational mechanisms for coordinating inter-subunit interactions with DNA cleavage. J Mol Biol. 2012;424: 109–124. doi: 10.1016/j.jmb.2012.07.014 22841979 10.1016/j.jmb.2012.07.014 PMC3584591

[24] Cook DE, Zdraljevic S, Tanny RE, Seo B, Riccardi DD, Noble LM, et al The Genetic Basis of Natural Variation in Caenorhabditis elegans Telomere Length. Genetics. 2016;204: 371–383. doi: 10.1534/genetics.116.191148 27449056 10.1534/genetics.116.191148 PMC5012401

[25] Rockman MV, Skrovanek SS, Kruglyak L. Selection at linked sites shapes heritable phenotypic variation in *C*. *elegans*. Science. 2010;330: 372–376. doi: 10.1126/science.1194208 20947766 10.1126/science.1194208 PMC3138179

[26] Schmidt BH, Osheroff N, Berger JM. Structure of a topoisomerase II-DNA-nucleotide complex reveals a new control mechanism for ATPase activity. Nat Struct Mol Biol. 2012;19: 1147–1154. doi: 10.1038/nsmb.2388 23022727 10.1038/nsmb.2388 PMC3492516

[27] Bandele OJ, Osheroff N. The efficacy of topoisomerase II-targeted anticancer agents reflects the persistence of drug-induced cleavage complexes in cells. Biochemistry. American Chemical Society; 2008;47: 11900–11908. doi: 10.1021/bi800981j 18922022 10.1021/bi800981j PMC2626429

[28] Deweese JE, Osheroff N. The DNA cleavage reaction of topoisomerase II: wolf in sheep’s clothing. Nucleic Acids Res. Oxford University Press; 2009;37: 738–748. doi: 10.1093/nar/gkn937 19042970 10.1093/nar/gkn937 PMC2647315

[29] Gao H, Huang K-C, Yamasaki EF, Chan KK, Chohan L, Snapka RM. XK469, a selective topoisomerase IIβ poison. Proceedings of the National Academy of Sciences. 1999;96: 12168–12173. doi: 10.1073/pnas.96.21.12168 10.1073/pnas.96.21.12168 PMC18430 10518594

[30] Bromberg KD, Burgin AB, Osheroff N. A Two-drug Model for Etoposide Action against Human Topoisomerase IIα. J Biol Chem. 2003;278: 7406–7412. doi: 10.1074/jbc.M212056200 12473657 10.1074/jbc.M212056200

[31] Koba M, Konopa J. [Actinomycin D and its mechanisms of action]. Postepy Hig Med Dosw. 2005;59: 290–298. Available: https://www.ncbi.nlm.nih.gov/pubmed/15995596 15995596

[32] Moen EL, Godley LA, Zhang W, Dolan ME. Pharmacogenomics of chemotherapeutic susceptibility and toxicity. Genome Med. 2012;4: 90 doi: 10.1186/gm391 23199206 10.1186/gm391 PMC3580423

[33] Giacomini KM, Brett CM, Altman RB, Benowitz NL, Dolan ME, Flockhart DA, et al The pharmacogenetics research network: from SNP discovery to clinical drug response. Clin Pharmacol Ther. 2007;81: 328–345. doi: 10.1038/sj.clpt.6100087 17339863 10.1038/sj.clpt.6100087 PMC5006950

[34] Huang RS, Duan S, Bleibel WK, Kistner EO, Zhang W, Clark TA, et al A genome-wide approach to identify genetic variants that contribute to etoposide-induced cytotoxicity. National Acad Sciences; 2007;104: 9758–9763. doi: 10.1073/pnas.0703736104 17537913 10.1073/pnas.0703736104 PMC1887589

[35] Low S-K, Chung S, Takahashi A, Zembutsu H, Mushiroda T, Kubo M, et al Genome-wide association study of chemotherapeutic agent-induced severe neutropenia/leucopenia for patients in Biobank Japan. Cancer Sci. 2013;104: 1074–1082. doi: 10.1111/cas.12186 23648065 10.1111/cas.12186 PMC7657179

[36] Lek M, Karczewski KJ, Minikel EV, Samocha KE, Banks E, Fennell T, et al Analysis of protein-coding genetic variation in 60,706 humans. Nature. 2016;536: 285–291. doi: 10.1038/nature19057 27535533 10.1038/nature19057 PMC5018207

[37] Chen SH, Chan N-L, Hsieh T-S. New mechanistic and functional insights into DNA topoisomerases. Annu Rev Biochem. 2013;82: 139–170. doi: 10.1146/annurev-biochem-061809-100002 23495937 10.1146/annurev-biochem-061809-100002

[38] Felix CA, Kolaris CP, Osheroff N. Topoisomerase II and the etiology of chromosomal translocations. DNA Repair. 2006;5: 1093–1108. doi: 10.1016/j.dnarep.2006.05.031 16857431 10.1016/j.dnarep.2006.05.031

[39] Cowell IG, Sondka Z, Smith K, Lee KC, Manville CM, Sidorczuk-Lesthuruge M, et al Model for MLL translocations in therapy-related leukemia involving topoisomerase IIβ-mediated DNA strand breaks and gene proximity. Proc Natl Acad Sci U S A. 2012;109: 8989–8994. doi: 10.1073/pnas.1204406109 22615413 10.1073/pnas.1204406109 PMC3384169

[40] Azarova AM, Lyu YL, Lin C-P, Tsai Y-C, Lau JY-N, Wang JC, et al Roles of DNA topoisomerase II isozymes in chemotherapy and secondary malignancies. Proc Natl Acad Sci U S A. National Acad Sciences; 2007;104: 11014–11019. doi: 10.1073/pnas.0704002104 17578914 10.1073/pnas.0704002104 PMC1904155

[41] Ratain MJ, Kaminer LS, Bitran JD, Larson RA, Le Beau MM, Skosey C, et al Acute nonlymphocytic leukemia following etoposide and cisplatin combination chemotherapy for advanced non-small-cell carcinoma of the lung. Blood. 1987;70: 1412–1417. Available: https://www.ncbi.nlm.nih.gov/pubmed/2822173 2822173

[42] Zhang S, Liu X, Bawa-Khalfe T, Lu L-S, Lyu YL, Liu LF, et al Identification of the molecular basis of doxorubicin-induced cardiotoxicity. Nat Med. 2012;18: 1639–1642. doi: 10.1038/nm.2919 23104132 10.1038/nm.2919

[43] Vejpongsa P, Yeh ETH. Topoisomerase 2β: A Promising Molecular Target for Primary Prevention of Anthracycline-Induced Cardiotoxicity. Clinical Pharmacology & Therapeutics. Wiley Online Library; 2014;95: 45–52. http://onlinelibrary.wiley.com/doi/10.1038/clpt.2013.201/full 10.1038/clpt.2013.201 24091715

[44] Yeh ETH, Bickford CL. Cardiovascular complications of cancer therapy: incidence, pathogenesis, diagnosis, and management. J Am Coll Cardiol. 2009;53: 2231–2247. doi: 10.1016/j.jacc.2009.02.050 19520246 10.1016/j.jacc.2009.02.050

[45] Mariani A, Bartoli A, Atwal M, Lee KC, Austin CA, Rodriguez R. Differential Targeting of Human Topoisomerase II Isoforms with Small Molecules. J Med Chem. 2015;58: 4851–4856. doi: 10.1021/acs.jmedchem.5b00473 25945730 10.1021/acs.jmedchem.5b00473

[46] Yang J, Bogni A, Schuetz EG, Ratain M, Dolan ME, McLeod H, et al Etoposide pathway. Pharmacogenet Genomics. 2009;19: 552–553. doi: 10.1097/FPC.0b013e32832e0e7f 19512958 10.1097/FPC.0b013e32832e0e7f PMC4164627

[47] Sim SC, Altman RB, Ingelman-Sundberg M. Databases in the area of pharmacogenetics. Hum Mutat. 2011;32: 526–531. doi: 10.1002/humu.21454 21309040 10.1002/humu.21454 PMC3352027

[48] Andersen EC, Bloom JS, Gerke JP, Kruglyak L. A variant in the neuropeptide receptor npr-1 is a major determinant of *Caenorhabditis elegans* growth and physiology. 2014;10: e1004156 doi: 10.1371/journal.pgen.1004156 24586193 10.1371/journal.pgen.1004156 PMC3937155

[49] Cook DE, Zdraljevic S, Roberts JP, Andersen EC. CeNDR, the Caenorhabditis elegans natural diversity resource. Nucleic Acids Res. 2016; doi: 10.1093/nar/gkw893 27701074 10.1093/nar/gkw893 PMC5210618

[50] Boyd WA, Smith MV, Freedman JH. Caenorhabditis elegans as a model in developmental toxicology. Methods Mol Biol. Totowa, NJ: Humana Press; 2012;889: 15–24. doi: 10.1007/978-1-61779-867-2_3 22669657 10.1007/978-1-61779-867-2_3 PMC3513774

[51] Shimko TC, Andersen EC. COPASutils: an R package for reading, processing, and visualizing data from COPAS large-particle flow cytometers. PLoS One. Public Library of Science; 2014;9: e111090 doi: 10.1371/journal.pone.0111090 25329171 10.1371/journal.pone.0111090 PMC4203834

[52] Brem RB, Kruglyak L. The landscape of genetic complexity across 5,700 gene expression traits in yeast. Proc Natl Acad Sci U S A. 2005;102: 1572–1577. doi: 10.1073/pnas.0408709102 15659551 10.1073/pnas.0408709102 PMC547855

[53] Kang HM, Zaitlen NA, Wade CM, Kirby A, Heckerman D, Daly MJ, et al Efficient Control of Population Structure in Model Organism Association Mapping. Genetics. 2008;178: 1709–1723. doi: 10.1534/genetics.107.080101 18385116 10.1534/genetics.107.080101 PMC2278096

[54] Endelman JB. Ridge Regression and Other Kernels for Genomic Selection with R Package rrBLUP. The Plant Genome Journal. Crop Science Society of America; 2011;4: 250–256. doi: 10.3835/plantgenome2011.08.0024

[55] Andersen EC, Bloom JS, Kruglyak L, Félix M-A, Ghosh R, Gerke JP, et al Chromosome-scale selective sweeps shape *Caenorhabditis elegans* genomic diversity. Nat Genet. Nature Publishing Group, a division of Macmillan Publishers Limited. All Rights Reserved.; 2012;44: 285–290. doi: 10.1038/ng.1050 22286215 10.1038/ng.1050 PMC3365839

[56] Cingolani P, Platts A, Wang LL, Coon M, Nguyen T, Wang L, et al A program for annotating and predicting the effects of single nucleotide polymorphisms, SnpEff: SNPs in the genome of Drosophila melanogaster strain w1118; iso-2; iso-3. Fly. 2012;6: 80–92. doi: 10.4161/fly.19695 22728672 10.4161/fly.19695 PMC3679285

[57] Kim H, Ishidate T, Ghanta KS, Seth M, Conte D, Shirayama M, et al A co-CRISPR strategy for efficient genome editing in Caenorhabditis elegans. Genetics. 2014;197: 1069–1080. doi: 10.1534/genetics.114.166389 24879462 10.1534/genetics.114.166389 PMC4125384

[58] Paix A, Folkmann A, Rasoloson D, Seydoux G. High Efficiency, Homology-Directed Genome Editing in Caenorhabditis elegans Using CRISPR-Cas9 Ribonucleoprotein Complexes. Genetics. 2015;201: 47–54. doi: 10.1534/genetics.115.179382 26187122 10.1534/genetics.115.179382 PMC4566275

[59] Jacobson MP, Friesner RA, Xiang Z, Honig B. On the role of the crystal environment in determining protein side-chain conformations. J Mol Biol. 2002;320: 597–608. doi: 10.1016/S0022-2836(02)00470-9 12096912 10.1016/s0022-2836(02)00470-9

[60] Jacobson MP, Pincus DL, Rapp CS, Day TJF, Honig B, Shaw DE, et al A hierarchical approach to all-atom protein loop prediction. Proteins. 2004;55: 351–367. doi: 10.1002/prot.10613 15048827 10.1002/prot.10613

[61] Davis IW, Leaver-Fay A, Chen VB, Block JN, Kapral GJ, Wang X, et al MolProbity: all-atom contacts and structure validation for proteins and nucleic acids. Nucleic Acids Res. 2007;35: W375–83. doi: 10.1093/nar/gkm216 17452350 10.1093/nar/gkm216 PMC1933162

[62] Sastry GM, Adzhigirey M, Day T, Annabhimoju R, Sherman W. Protein and ligand preparation: parameters, protocols, and influence on virtual screening enrichments. J Comput Aided Mol Des. 2013;27: 221–234. doi: 10.1007/s10822-013-9644-8 23579614 10.1007/s10822-013-9644-8

