# Supplementary figures and images for: Natural variation in a single amino acid substitution underlies physiological responses to topoisomerase II poisons
Source: PLoS Genet. 2017 Jul 12;13(7):e1006891. doi: 10.1371/journal.pgen.1006891 (PMC5529024; doi:10.1371/journal.pgen.1006891)

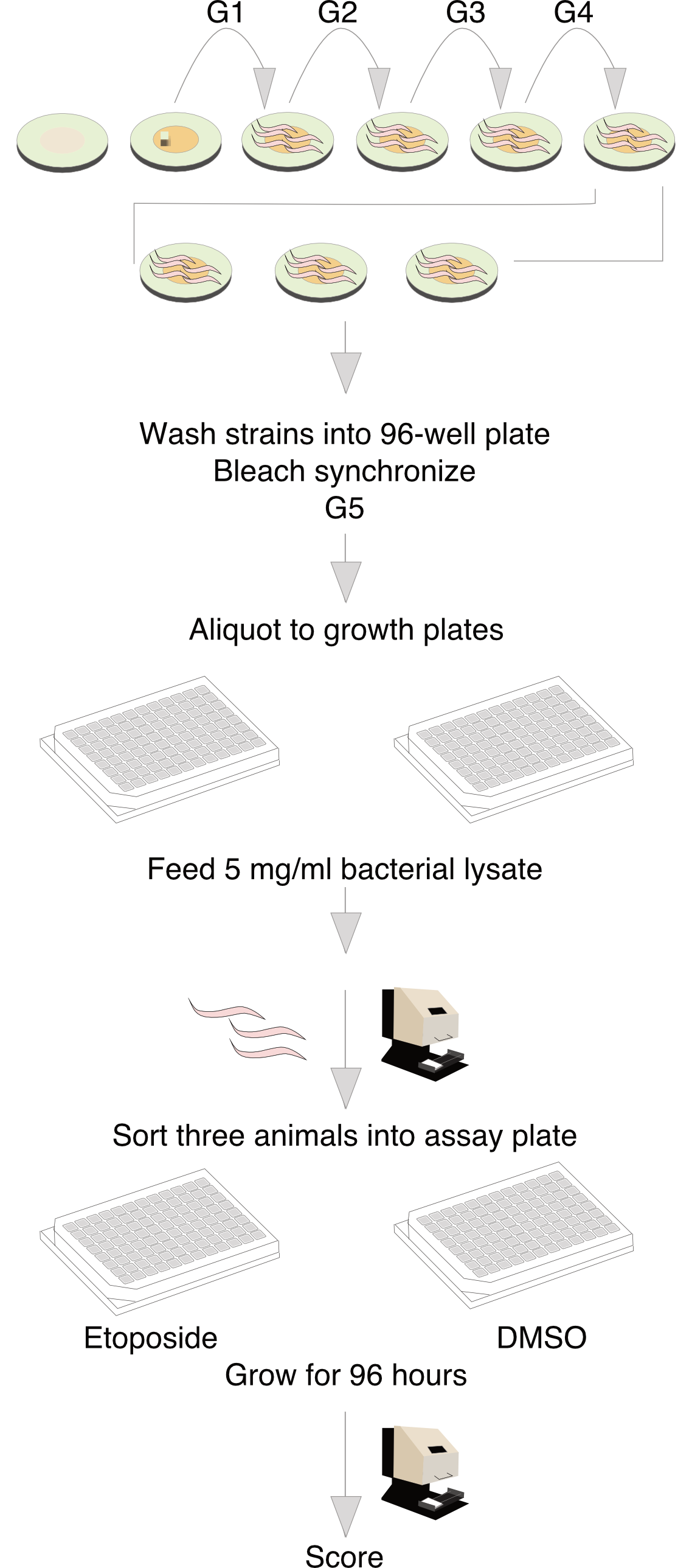

Supplement: S1 Fig — Individual strains are passaged for four generations on agar plates seeded with OP50 bacteria by transfer of seven L4 larval stage animals to a fresh plate each generation every three days. Animals are bleach synchronized and aliquoted to 96-well microtiter plates in 50 μl of K medium at a concentration of one embryo per μL. Aliquoted embryos are incubated overnight at 20°C. The following day, 5 μl of 50 mg/ml HB101 lysate is added to each well. Animals are then grown for two days to the L4 larval stage. Then, three L4 animals are sorted to assay plates containing drug or DMSO using the BIOSORT. Four days later, 200 μl M9 plus 50 mM sodium azide is added to each well and strains are scored using the BIOSORT. (TIFF) [file pgen.1006891.s001.tiff]

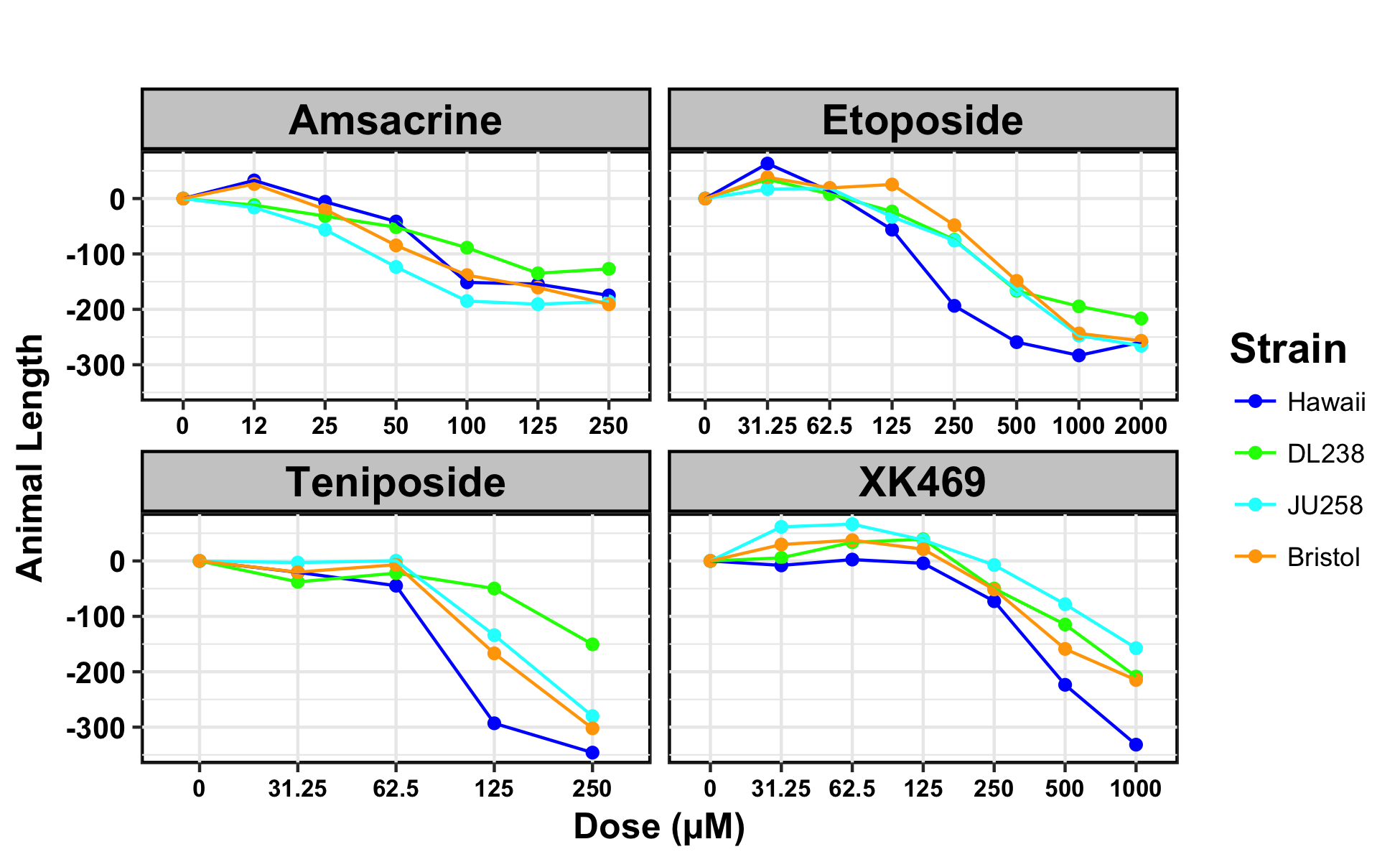

Supplement: S2 Fig — For each drug, concentration in μM is plotted on the x-axis, and the regressed 75th quantile of animal length is plotted on the y-axis. The red numbers above the dose response lines correspond to broad-sense heritability estimates. The heritability estimates for the drug concentrations used in experiments throughout this study are: H2 = 0.47 for amsacrine at 50 μM, H2 = 0.62 for etoposide at 250 μM, H2 = 0.73 for teniposide at 125 μM, and H2 = 0.9 for XK469 at 1000 μM. The Bristol strain (N2) is colored in orange, the Hawaiian strain (CB4856) in blue, JU258 in cyan, and DL238 in green. (TIFF) [file pgen.1006891.s002.tiff]

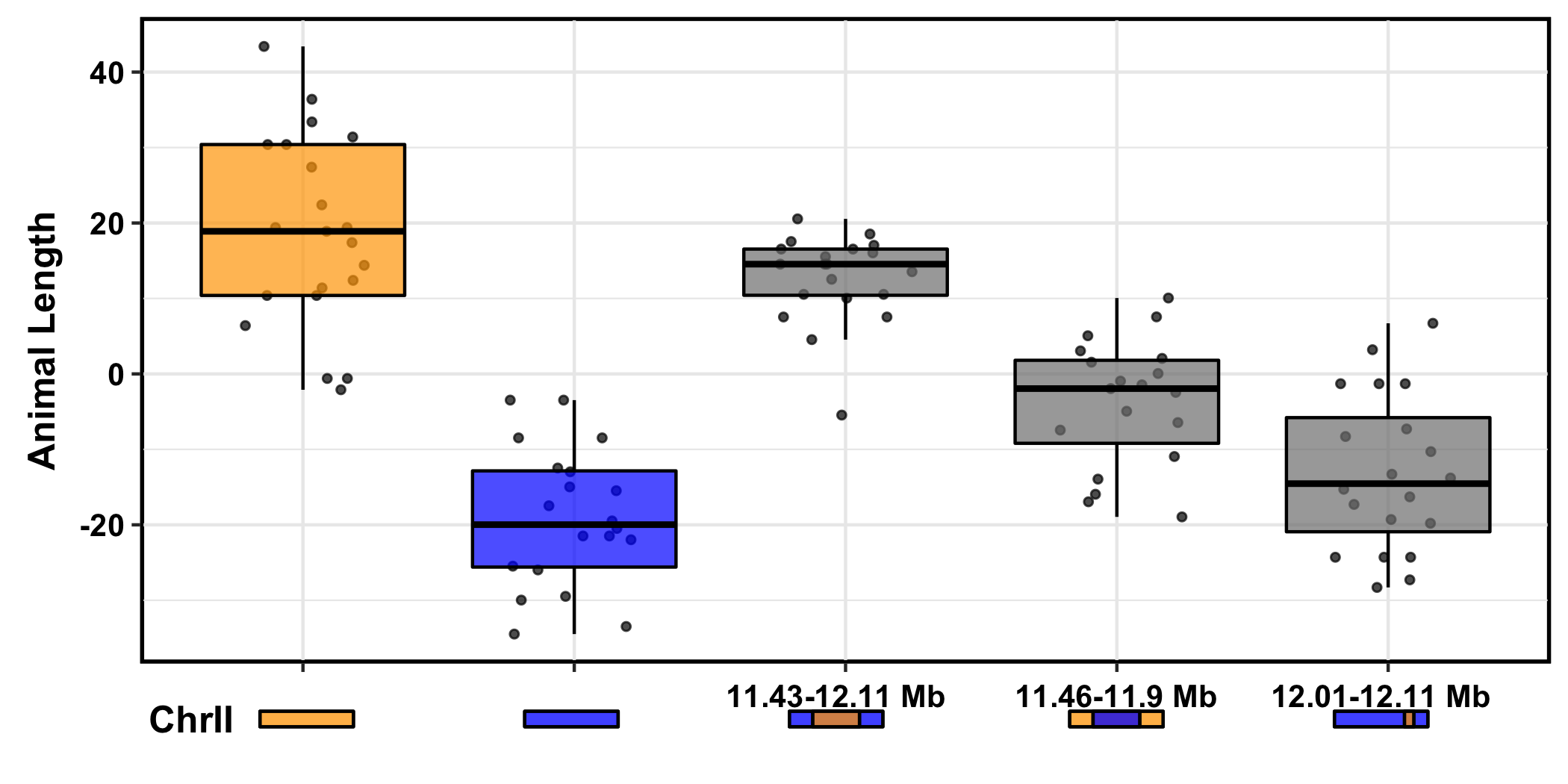

Supplement: S3 Fig — Tukey box plots of NIL regressed median animal length in the presence of etoposide are shown. NIL genotypes are indicated below the plot by colored rectangles, Bristol (orange) or Hawaii (blue). Numbers above rectangles correspond to the genomic position of the introgression region on chromosome II. (TIFF) [file pgen.1006891.s003.tiff]

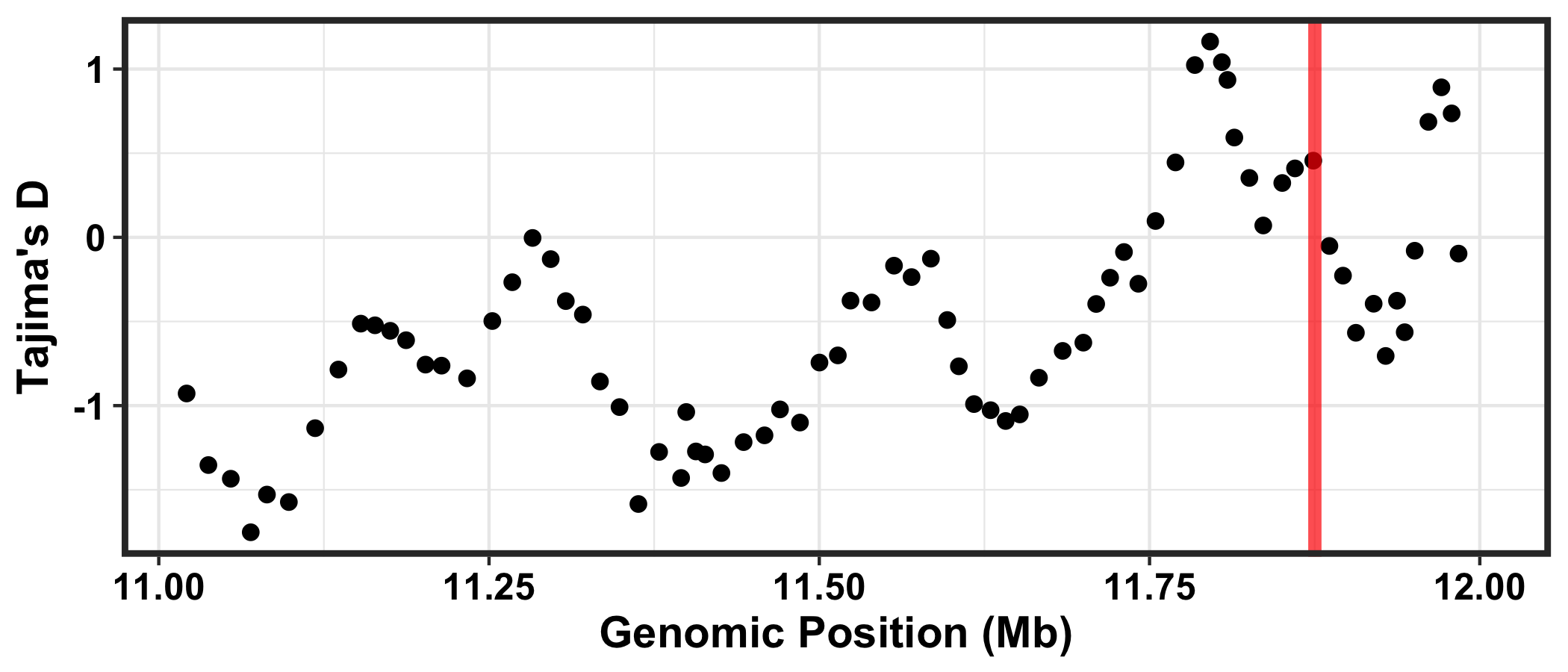

Supplement: S4 Fig — Divergence, as measured by Tajima's D, is shown across the etoposide QTL confidence interval (II:11021073–12008179). The whole-genome SNV data set [24,49] was used for Tajima’s D calculations. Window size for the calculations was 300 SNVs with a 100 SNV sliding window size. The vertical red line marks the position of the top-2 locus. The tajimas_d function in the cegwas package was used to perform the calculations. (TIFF) [file pgen.1006891.s004.tiff]

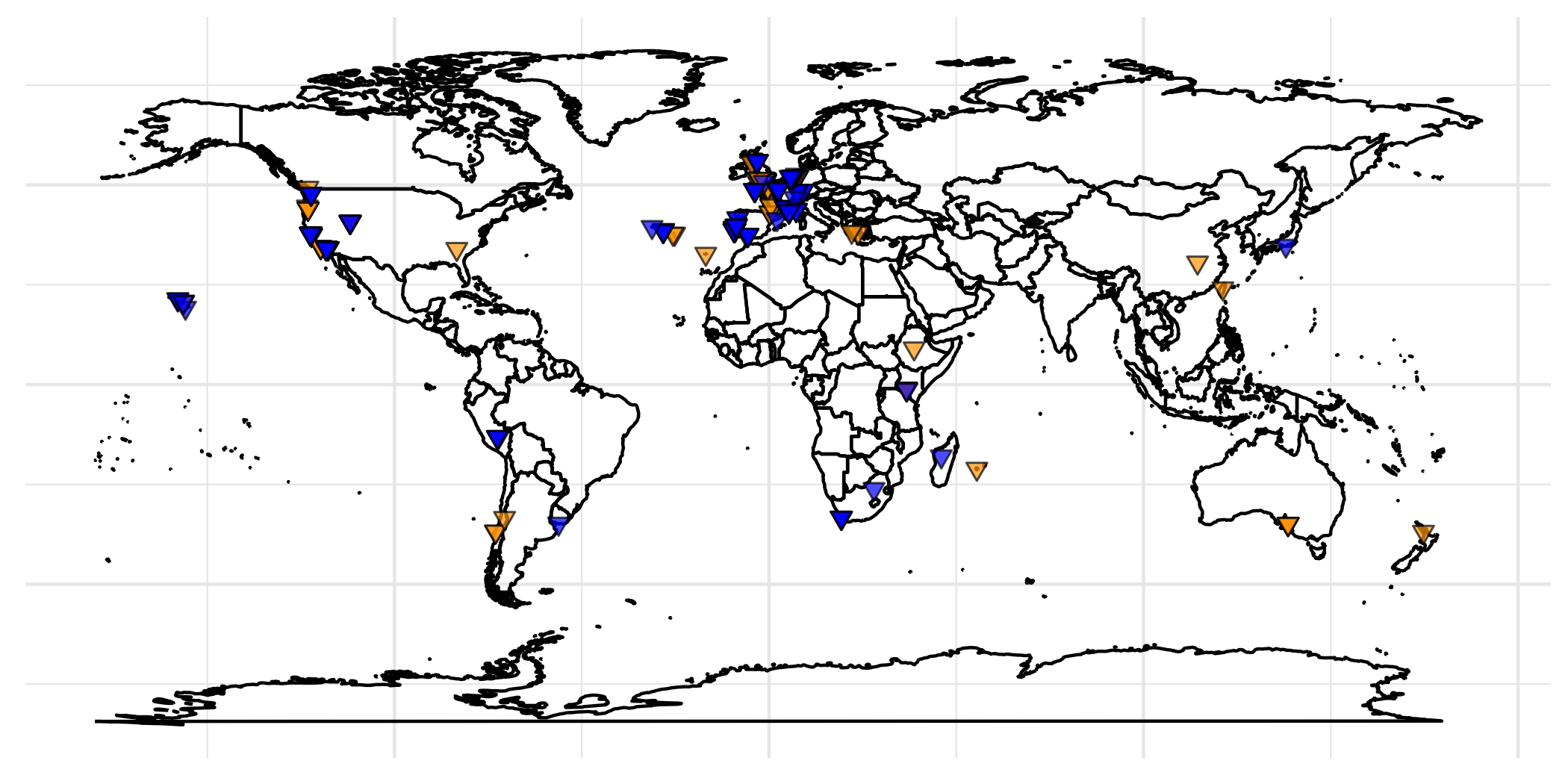

Supplement: S5 Fig — Glutamine (REF) is shown in orange and methionine (ALT) is shown in blue. Latitude and longitude coordinates of sampling locations were used to plot individual strains on the map [49]. (TIFF) [file pgen.1006891.s005.tiff]

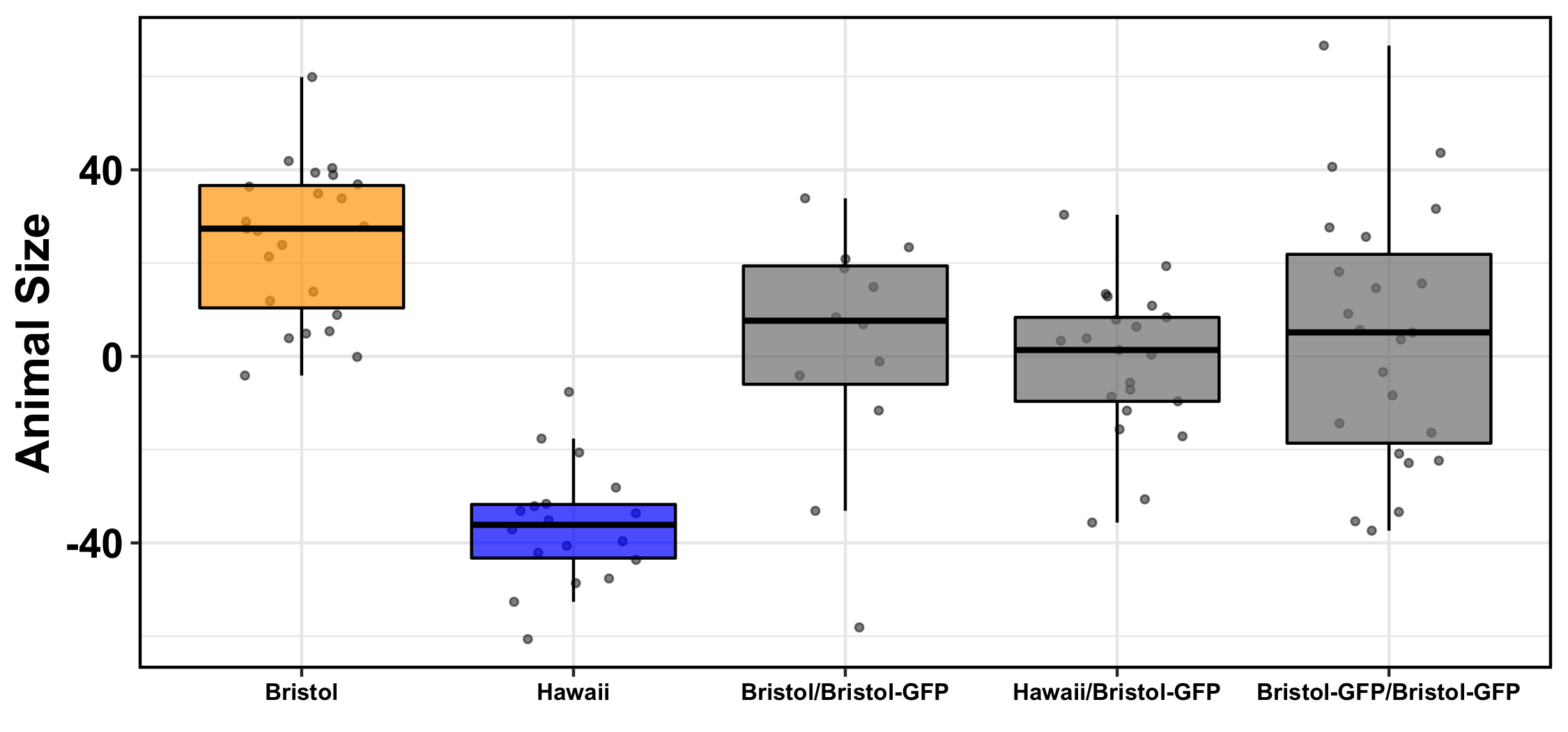

Supplement: S6 Fig — Tukey box plots of Bristol and Hawaii regressed median animal length in response to etoposide are plotted in orange and blue, respectively. GFP-containing Bristol strains (EG7952) were crossed to the Bristol and Hawaii strains and heterozygous progeny were assayed using the high-throughput fitness assay. Tukey box plots of heterozygotes are shown in gray. All heterozygote strains are significantly different from the parental Bristol and Hawaii strains (Tukey’s HSD, p < 0.02). All heterozygote strains are not significantly different from each other (Tukey’s HSD, p > 0.91). (TIFF) [file pgen.1006891.s006.tiff]

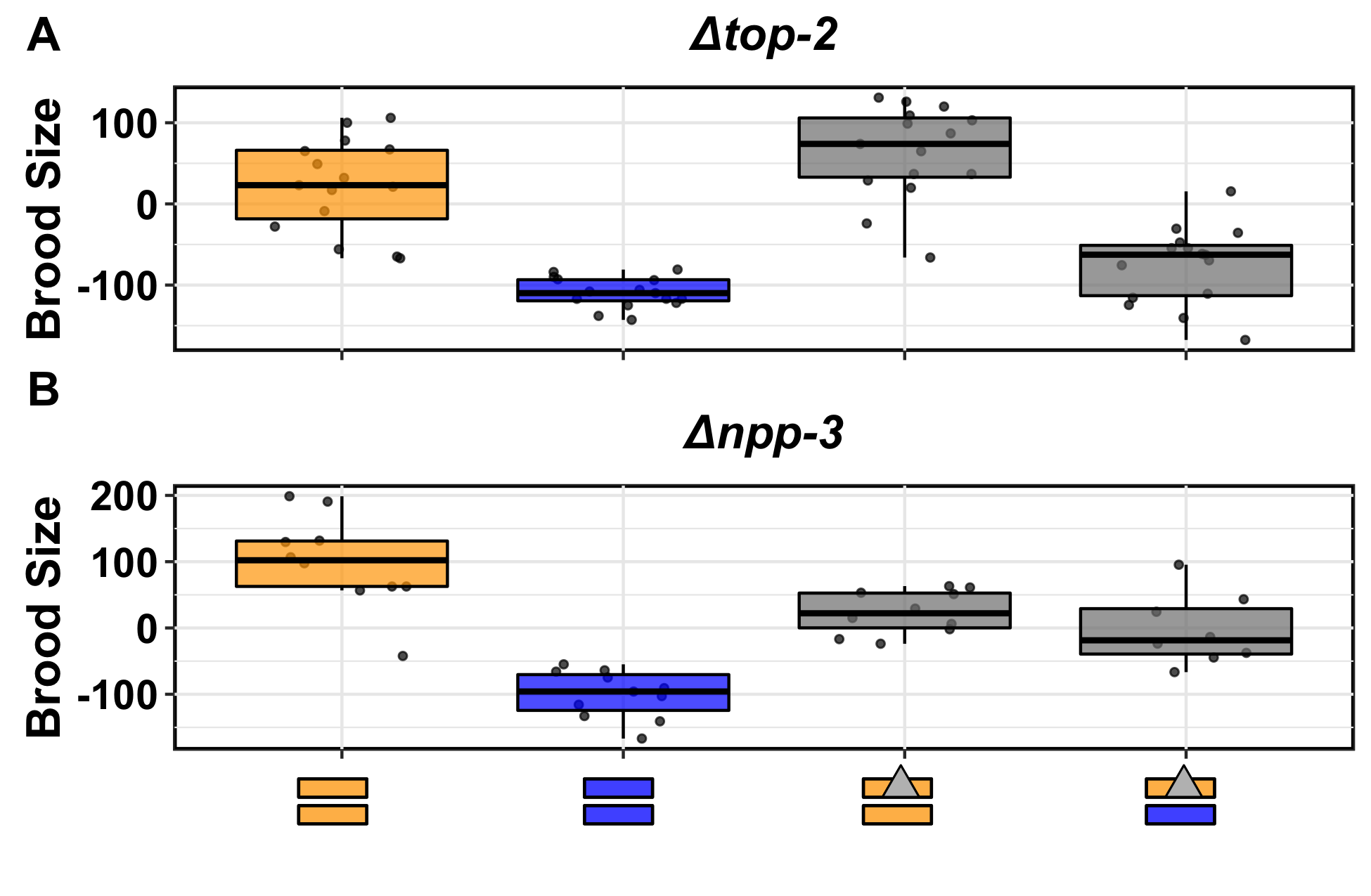

Supplement: S7 Fig — Tukey box plots of the residual brood size distribution of Bristol (orange) and Hawaii (blue) compared to two heterozygous (A) top-2 and (B) npp-3 deletion strains (gray) in response to etoposide are plotted. Orange (Bristol) and blue (Hawaii) rectangles below the plot correspond to the two chromosome II homolog genotypes. Gray triangles denote chromosomes with the top-2 deletion allele. (A) The Bristol strain and the Bristol/Bristol(Δtop-2) heterozygous strain are not significantly different from each other (Tukey’s HSD, p-value 0.104). The Hawaii strain and the Hawaii/Bristol(Δtop-2) heterozygous strain are not significantly different from each other (Tukey’s HSD, p-value 0.226). All other comparisons are significant (Tukey’s HSD p-value < 4.1E-6). (B) The Hawaii/Bristol(Δnpp-3) and the Bristol/Bristol(Δnpp-3) heterozygous strain are not significantly different from each other (Tukey’s HSD, p-value 0.68), but all other comparisons are significant (Tukey’s HSD p-value < 0.009). (TIFF) [file pgen.1006891.s007.tiff]

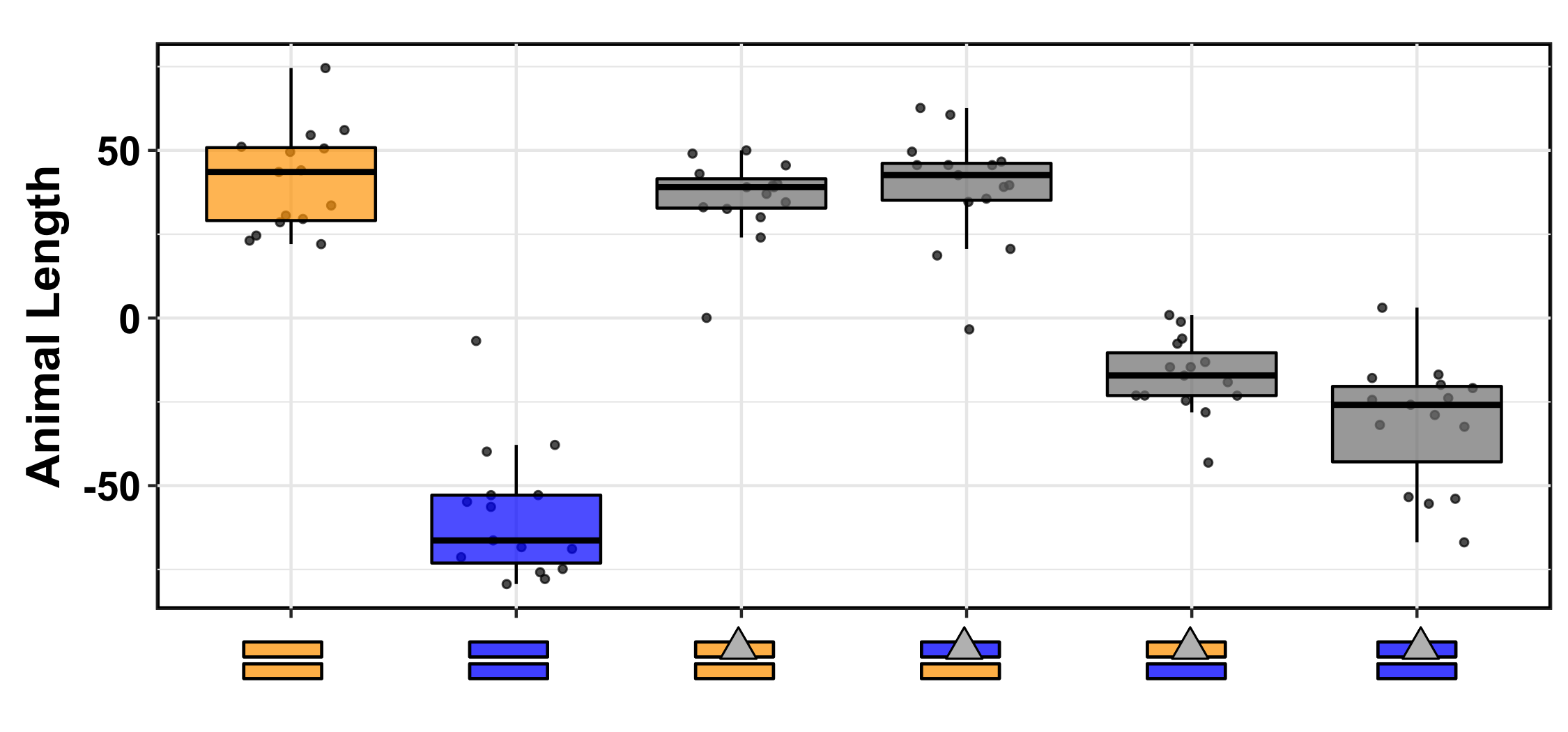

Supplement: S8 Fig — (A) Tukey box plots of the residual median animal length distribution of the Bristol (orange) and Hawaii (blue) strains compared to four heterozygous top-2 deletion strains (gray) in response to etoposide are plotted. Orange (Bristol) and blue (Hawaii) rectangles below the plot correspond to the two chromosome II homolog genotypes. Gray triangles denote chromosomes with the top-2 deletion allele. Phenotypes of heterozygous deletion strains with the Bristol TOP-2 allele are not significantly different from the parental Bristol strain. The Hawaii/Bristol(Δtop-2) and the Hawaii/Hawaii(Δtop-2) strain phenotypes are not significantly different from each other (Tukey’s HSD, p-value = 0.16), but are both significantly different from the parental Hawaii strain (Tukey’s HSD, p-value < 1E-7 and p-value = 0.0001, respectively). (TIFF) [file pgen.1006891.s008.tiff]

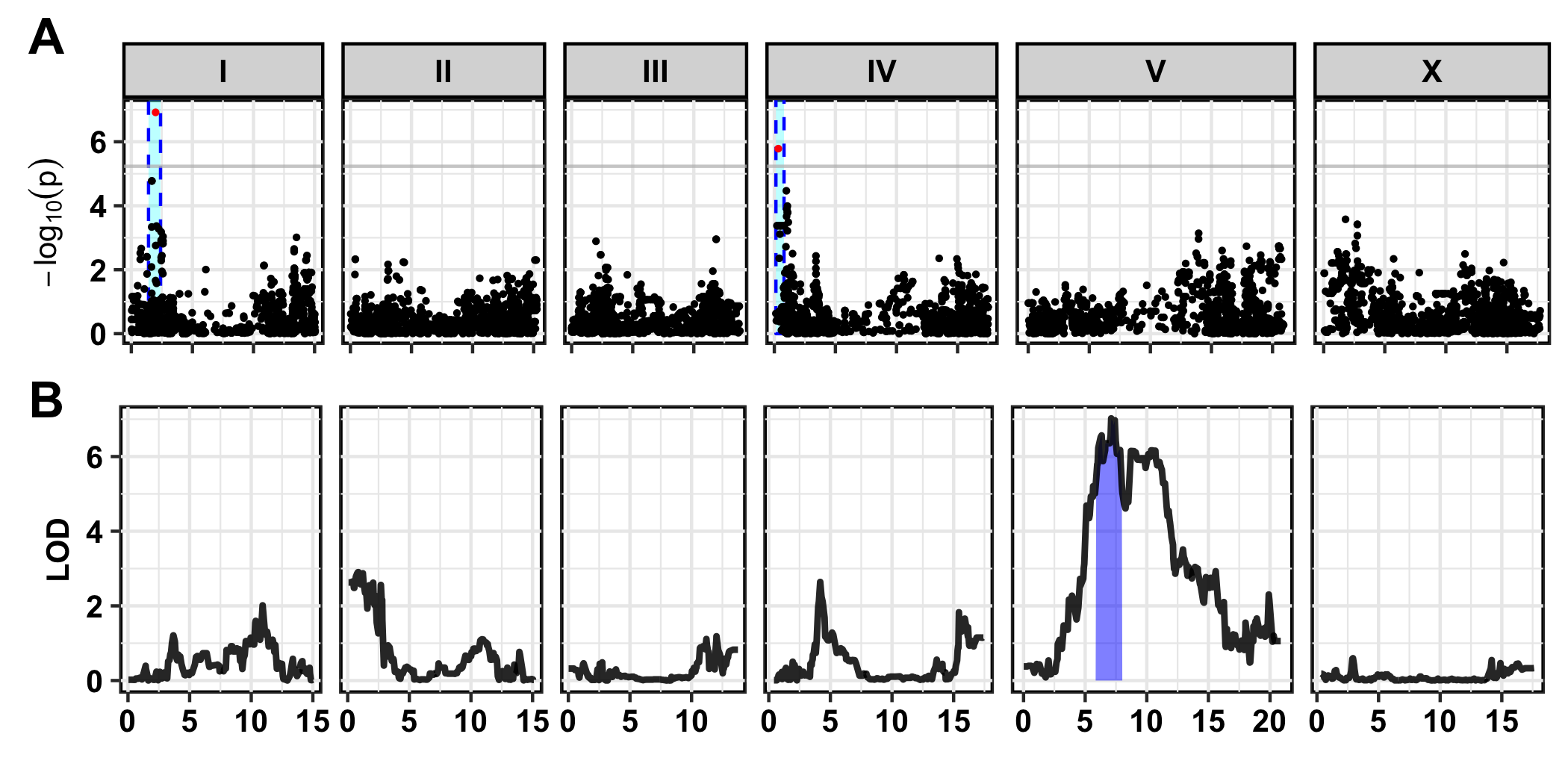

Supplement: S9 Fig — (A) A manhattan plot for regressed fraction of animals in the L1 larval stage in the presence of amsacrine is plotted. Each dot represents an SNV that is present in at least 5% of the phenotyped population. The –log10(p) for each SNV is plotted on the y-axis and the genomic position (Mb) is on the x-axis. Each tick on the x-axis corresponds to 5 Mb. SNVs are colored red if they pass the genome-wide Bonferroni-corrected significance threshold, which is denoted by the gray horizontal line. The genomic regions of interest are represented by cyan rectangles surrounding each QTL. (B) A linkage mapping plot for regressed fraction of animals in the L1 larval stage in the presence of amsacrine is shown. The significance value (logarithm of odds, LOD, ratio) is plotted on the y-axis and genomic position (Mb), separated by chromosome, on the x-axis. Each tick on the x-axis corresponds to 5 Mb. The associated 1.5 LOD-drop confidence intervals are represented by blue bars. (TIFF) [file pgen.1006891.s009.tiff]

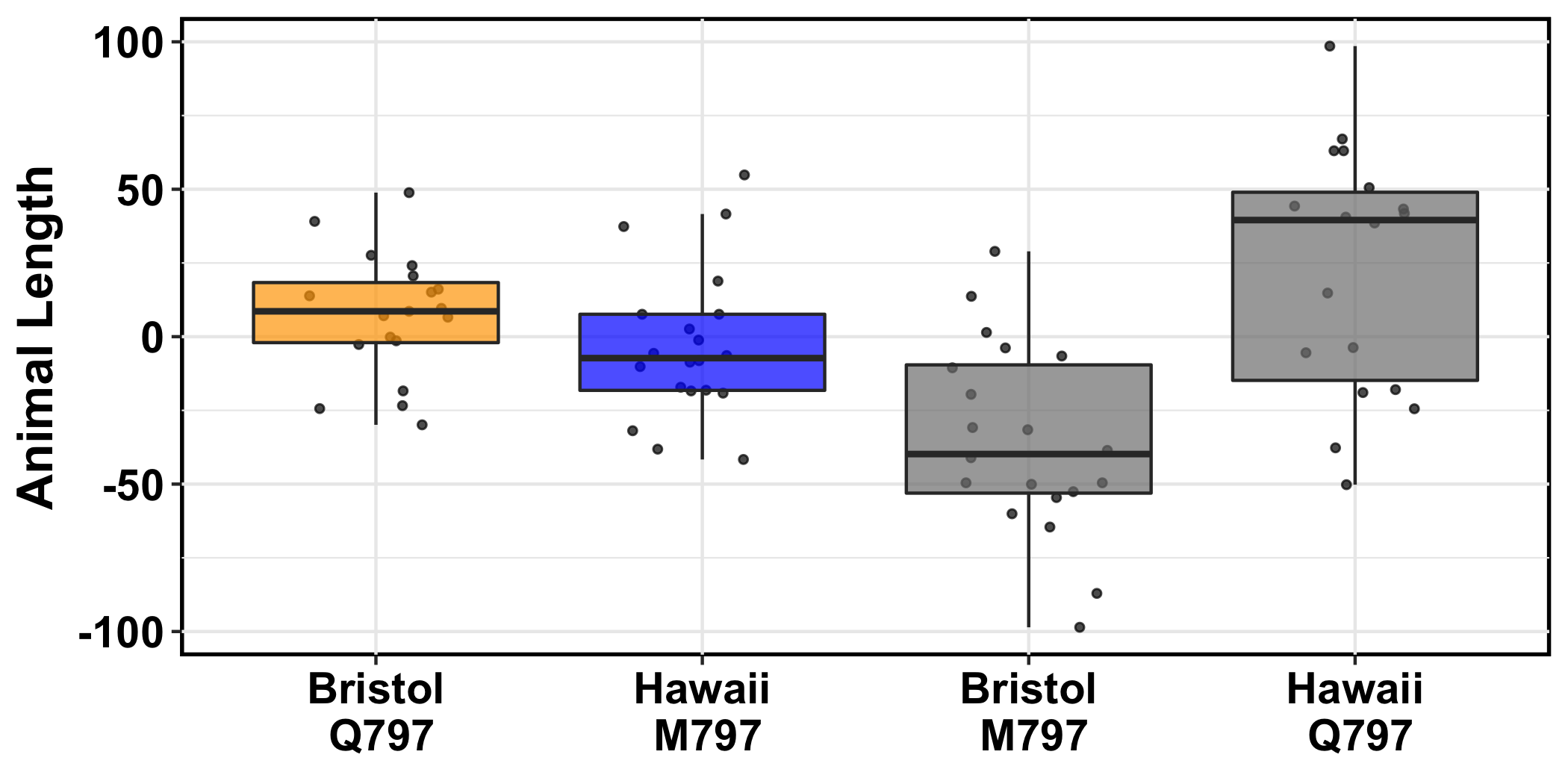

Supplement: S10 Fig — Tukey box plots of regressed median animal length in response to XK469 are plotted. Orange corresponds to the Bristol genetic background and blue to Hawaii. Allele-replacement strains are denoted by their genetic background and the corresponding residue change at position 797 of TOP-2. Strains containing the glutamine TOP-2 allele are more resistant to XK469 than strains with the methionine allele. All strain comparisons are significantly different from each other (Tukey’s HSD, p-value < 0.01) (TIFF) [file pgen.1006891.s010.tiff]

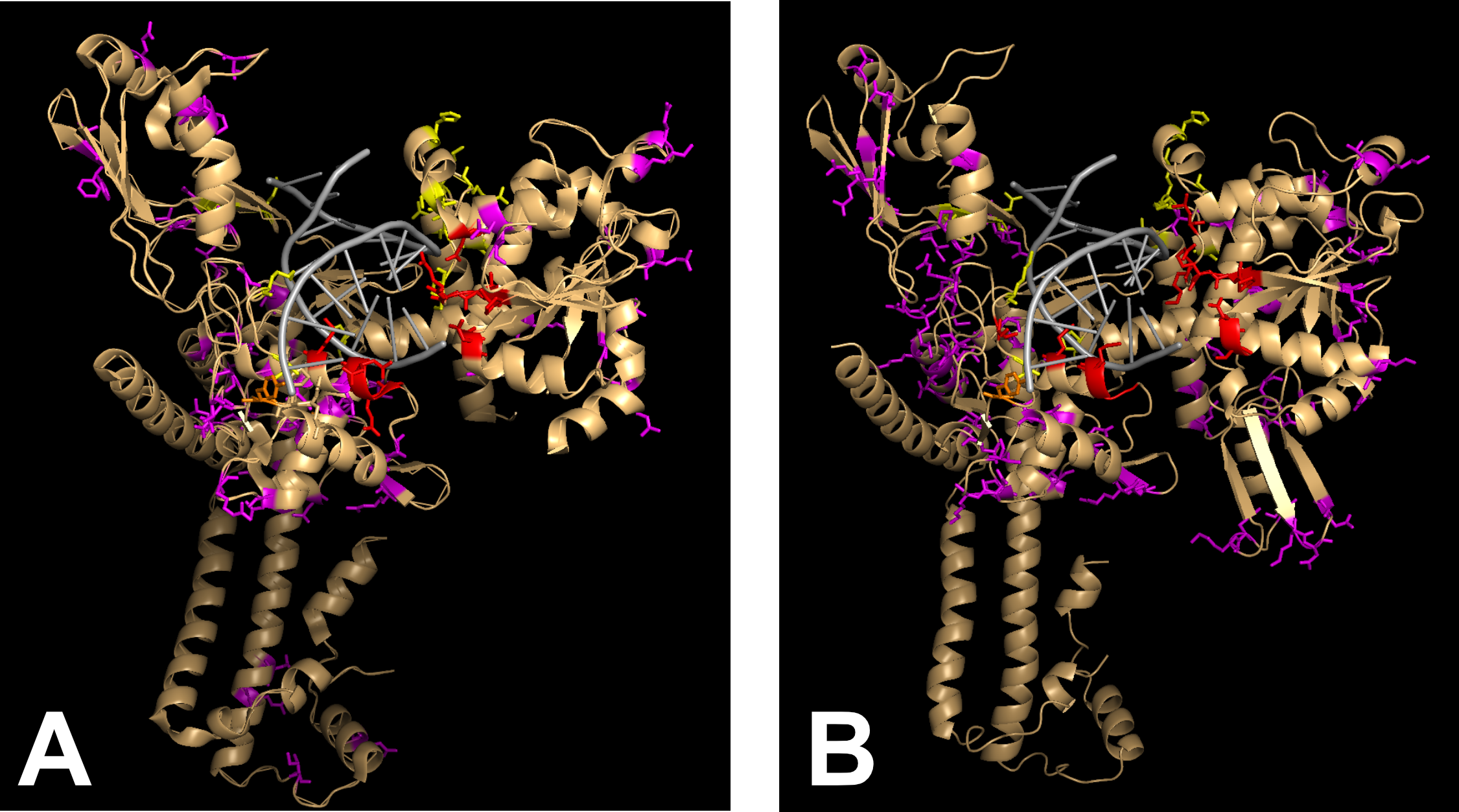

Supplement: S11 Fig — (A) hTOPOIIα (PDB 4FM9) and (B) hTOPIIβ (PDB 3QX3) are shown. Putative etoposide-binding residues are highlighted in red, DNA-binding residues are highlighted in yellow, and the catalytic tyrosine is highlighted in orange. The cartoon representation of the peptide backbones is shown in tan, and DNA is shown in gray. Residues that vary in the human population are highlighted in purple. (TIFF) [file pgen.1006891.s011.tiff]
