# Supplementary material for: Natural variation in a single amino acid substitution underlies physiological responses to topoisomerase II poisons
Source: PLoS Genet. 2017 Jul 12;13(7):e1006891. doi: 10.1371/journal.pgen.1006891 (PMC5529024; doi:10.1371/journal.pgen.1006891)
Supplement: S1 Supplemental Methods — (DOCX) [file pgen.1006891.s030.docx]

**Supplemental Methods:**

**Generation of NIL strains**

RIAILs that switched genotypes near indel markers were used as starting strains to generate all NILs used in this study. The RIAIL QX327 was used to generate ECA219 *eanIR139[II, CB>N2]*, QX322 was used to generate ECA216 *eanIR136[II, N2>CB4856]*, and QX327 was used to generate ECA220 *eanIR140[II, N2>CB4856]*. Primers oECA605, oECA606, oECA607, and oECA608 were used to follow two indels at 11.64 and 11.91Mb on chromosome II to generate the Hawaii NIL ECA219. Primers oECA593, oECA594, oECA595, and oECA596 were used to follow two indels at 11.43 and 12.11Mb on chromosome II to generate the Bristol NIL ECA216. Primers oECA601, oECA602, oECA03, and oECA604 were used to follow two indels at 12.01 and 12.1Mb on chromosome II to generate the Bristol NIL ECA220. For each NIL, eight crosses were performed followed by six generations of selfing to homozygose the genome. All PCRs were multiplexed and performed with four primers at an annealing temperature of 58ºC for 34 cycles with Taq polymerase.

**Generation of Hawaii *∆top-2* strain**

Heterozygous Bristol *top-2(ok1930)* and Bristol *mIn1* strains were then crossed to the Hawaiian strain. Males from these crosses were used to outcross to CB4856 for 10 consecutive generations. The primers oECA1003 and oECA1004 were used to follow the *top-2(ok1930)* allele during the back crosses. *mIn1* crosses were followed by identifying green fluorescent male progeny. At the end of 10 generations, the CB4856 crosses to *top-2(ok1930)* and *mIn1* were crossed to each other to generate a *top-2(ok1930)/mIn1* strain in the CB4856 genetic background and named ECA338.

**Generation of top-2 allele replacement strains**

All allele replacement strains were generated using CRISPR/Cas9-mediated genome engineering, using the co-CRISPR approach described in the main text methods (Kim *et al.* 2014) with Cas9 ribonucleoprotein delivery (Paix *et al.* 2015). The resulting Bristol swap strains were named ECA401 *top-2(ean2[Q797M])* and ECA402 *top-2(ean3[Q797M])*. The resulting Hawaii strains were named ECA547 *top-2(ean4[M797Q])*, ECA548 *top-2(ean5[M797Q])*, and ECA549 *top-2(ean6[M797Q])*.

**Primer Table**

| **Name** | **Sequence** | **Description** |
| --- | --- | --- |
| **oECA593** | CCGGTGTTTCAGGGCAATTT | Generate NIL for etoposide interval 11470951-12115727 using RIAIL QX322 |
| **oECA596** | GCTACGGAATGTGCTGCTAC | Generate NIL for etoposide interval 11470951-12115727 using RIAIL QX322 |
| **oECA601** | GAAGTTTCGGGTCAATGTATCCA | Generate NIL for etoposide interval 11470951-12115727 using RIAIL QX327 |
| **oECA604** | GCTACGGAATGTGCTGCTAC | Generate NIL for etoposide interval 11470951-12115727 using RIAIL QX327 |
| **oECA605** | ATGCAACGTTTGACTGGCAT | Generate NIL for etoposide interval 11470951-12115727 using RIAIL QX103 |
| **oECA608** | CCATTGAATTAGTTGGCGGC | Generate NIL for etoposide interval 11470951-12115727 using RIAIL QX103 |
| **oECA1087** | GACGAGTACCATTGGAATAATCGGG | Verification CRISPR allele swap at top-2 Q778M variant |
| **oECA1124** | GGGAGAAGAAGGACCGAAAGC | Verification CRISPR allele swap at *top-2* Q778M variant |
| **oECA1003** | TCCAATCAAAGGATTCGAGG | 5’ primer used to verify presence of *K12D12.1(ok1930)* |
| **oECA1004** | ATGTCCTGGCCTTCCTTTTT | 3’ primer used to verify presence of *K12D12.1(ok1930)* |
| **dpy-10 repair** | CACTTGAACTTCAATACGGCAAGATGAGAATGACTGGAAACCGTACCGCATGCGGTGCCTATGGTAGCGGAGCTTCACATGGCTTCAGACCAACAGCCTAT | Repair template to generate dpy-10(cn64) mutation |
| **CB4856 Repair** | ACAGCGGAAGGTTCTCTTCGCGTGCTTCAAGAGAGCAGACAAGCGTGAAGTCAAAGTAGCTCAATTGGCTGGAGCTGTCGCTGAAATTTCTGCTTA**TCA**TCACGGAGAA***CAG***TCGCTTATGGGAACAATTGTGAATCTCG | Repair template to generate CB4856 M778Q allele swap. Green represents the gRNA sequence, red represents the PAM site, purple is the mutated nucleotide of the PAM site, and bold-underline-italic encodes for the desired edit. |
| **N2 Repair** | ACAGCGGAAGGTTCTCTTCGCGTGCTTCAAGAGAGCAGACAAGCGTGAAGTCAAAGTAGCTCAATTGGCTGGAGCTGTCGCTGAAATTTCTGCTTA**TCA**TCACGGAGAA***ATG***TCGCTTATGGGAACAATTGTGAATCTCG | Repair template to generate N2 Q778M allele swap. Green represents the gRNA sequence, red represents the PAM site, purple is the mutated nucleotide of the PAM site, and bold-underline-italic encodes for the desired edit. |
| ***top-2* crRNA CB4856** | TAAGCGACATTTCTCCGTGA | Guide sequence for crRNA to target *top-2* at M778 in CB4856 |
| ***top-2* crRNA N2** | TAAGCGACTGTTCTCCGTGA | Guide sequence for crRNA to target *top-2* at Q778 in N2 |
| ***dpy-10* crRNA** | GCTACCATAGGCACCACGAG | Guide sequence for crRNA to target dpy-10 |
| **TOP2A crRNA** | GTCATCATTAGTGACATCTG | targets sense strand of human TOP2A |
| **TOP2B crRNA** | GCTTGCTATACAACAGAAGA | targets sense strand of human TOP2B |
| **TOP2A M>Q (sense strand)** | AGTAAAAGCCTCAGCTTAATGAATCTTTTTTTCTCTACAG**CAA**TCACTAATGATGACCATTATCAATTTGGCTCAGAATTTTG | Repair template sequences (edited bases are shown in red, with the amino-acid changing mutations bolded, and the sgRNA binding site underlined) |
| **TOP2B M>Q (antisense strand)** | CAAAGTTCTGAGCTAAGTTCACAATAGTCATCATCAATGC**CAT**CTATACAACAGAAGAAGACAGAACATAACATTAATATTCT | Repair template sequences (edited bases shown in red, with the amino-acid changing mutations bolded, and the sgRNA binding site underlined) |
| **TOP2A forward** | TTGTGGAAAGGACGAAACACCGGTGAGGTTAAGTCATAATGTATTTGT | PCR primers, first round. Underlined sequence binds to target DNA. Red nucleotides are sequence adaptors used for the second round of PCR |
| **TOP2A reverse** | TCTACTATTCTTTCCCCTGCACTGTCCCCTGGCCTCTGCCACTAGAT | PCR primers, first round. Underlined sequence binds to target DNA. Red nucleotides are sequence adaptors used for the second round of PCR |
| **TOP2B forward** | TTGTGGAAAGGACGAAACACCGCTTTATTCTTCACTTGGATTTTAATTC | PCR primers, first round. Underlined sequence binds to target DNA. Red nucleotides are sequence adaptors used for the second round of PCR |
| **TOP2B reverse** | TCTACTATTCTTTCCCCTGCACTGTCCACAGCTATAATTCCATCGAACA | PCR primers, first round. Underlined sequence binds to target DNA. Red nucleotides are sequence adaptors used for the second round of PCR |
| **P5/forward prime** | AATGATACGGCGACCACCGAGATCTACACTCTTTCCCTACACGACGCTCTTCCGATCT[s]TTGTGGAAAGGACGAAACACCG | Green: P5 & P7 sequences for attachment to Illumina flow cell  Blue: Illumina sequencing primer binding sites  [s]: Stagger sequence. To prevent monotemplate reads, a mixture of eight unique oligonucleotides are used that vary in their length in this region. The nucleotides used in this position are: [no additional bases, C, GC, AGC, CAAC, TGCACC, ACGCAAC, GAAGACCC]  Red: Sequences that bind to round 1 PCR products  NNNNNNNN: 8 nucleotide barcode used to identify each sample |
| **P7/reverse primer** | CAAGCAGAAGACGGCATACGAGATNNNNNNNNGTGACTGGAGTTCAGACGTGTGCTCTTCCGATCTTCTACTATTCTTTCCCCTGCACTGT | Same as above |

**Strain table:**

| **Name** | **Allele Name** | **Genotype** |
| --- | --- | --- |
| **ECA215** | *eanIR135* | N2 (II:11.64 - 11.91 Mb) |
| **ECA216** | *eanIR136* | N2 (II:11.43 - 12.11 Mb) |
| **ECA219** | *eanIR139* | CB4856 (II:11.64 - 11.9 Mb) |
| **ECA220** | *eanIR140* | N2 (II:12.01 - 12.1 Mb) |
| **ECA338** | *K12D12.1(ok1930)* | (*K12D12.1(ok1930)/mIn1 [mIs14 dpy-10(e128)]* II) |
| **VC1474** | *K12D12.1(ok1930)* | (*K12D12.1(ok1930)/mIn1 [mIs14 dpy-10(e128)]* II) |
| **EG7952** | NA | [*oxTi207 [eft-3p::GFP::unc-54 3'UTR + hsp::peel-1 + NeoR + Cbr-unc-119(+)]]* |
| **ECA401** | top-2(*ean2*[Q797M] | N2 TOP2(Q797M) |
| **ECA402** | top-2(*ean3*[Q797M] | N2 TOP2(Q797M) |
| **ECA547** | top-2(*ean4*[M797Q]) | CB4856 TOP2(M797Q) |
| **ECA548** | top-2(*ean5*[M797Q]) | CB4856 TOP2(M797Q) |
| **ECA549** | top-2(*ean6*[M797Q]) | CB4856 TOP2(M797Q) |
